# Anti-estrogen Resistance in Human Breast Tumors Is Driven by JAG1-NOTCH4-Dependent Cancer Stem Cell Activity

**DOI:** 10.1016/j.celrep.2015.08.050

**Published:** 2015-09-17

**Authors:** Bruno M. Simões, Ciara S. O’Brien, Rachel Eyre, Andreia Silva, Ling Yu, Aida Sarmiento-Castro, Denis G. Alférez, Kath Spence, Angélica Santiago-Gómez, Francesca Chemi, Ahmet Acar, Ashu Gandhi, Anthony Howell, Keith Brennan, Lisa Rydén, Stefania Catalano, Sebastiano Andó, Julia Gee, Ahmet Ucar, Andrew H. Sims, Elisabetta Marangoni, Gillian Farnie, Göran Landberg, Sacha J. Howell, Robert B. Clarke

**Affiliations:** 1 Breast Cancer Now Research Unit, Institute of Cancer Sciences, University of Manchester, Wilmslow Road, Manchester M20 4BX, UK; 2 Department of Pharmacy, Health and Nutritional Sciences, University of Calabria, 87036 Arcavacata di Rende, Cosenza, Italy; 3 Faculty of Life Sciences, University of Manchester, Oxford Road, Manchester M13 9PT, UK; 4 Manchester Academic Health Science Centre, University Hospital of South Manchester NHS Foundation Trust, Southmoor Road, Manchester M23 9LT, UK; 5 Department of Surgery, Clinical Sciences, Lund University, Skåne University Hospital, 21428 Malmö, Sweden; 6 Cardiff School of Pharmacy and Pharmaceutical Sciences, University of Cardiff, Cardiff, Wales CF10 3NB, UK; 7 Applied Bioinformatics of Cancer Group, Systems Medicine Building, Western General Hospital, University of Edinburgh, Edinburgh EH4 2XU, UK; 8 Laboratoire d’Investigation Préclinique, Institut Curie, 26 rue d’Ulm 75248 Paris Cedex 05, France

## Abstract

Breast cancers (BCs) typically express estrogen receptors (ERs) but frequently exhibit de novo or acquired resistance to hormonal therapies. Here, we show that short-term treatment with the anti-estrogens tamoxifen or fulvestrant decrease cell proliferation but increase BC stem cell (BCSC) activity through JAG1-NOTCH4 receptor activation both in patient-derived samples and xenograft (PDX) tumors. In support of this mechanism, we demonstrate that high ALDH1 predicts resistance in women treated with tamoxifen and that a NOTCH4/HES/HEY gene signature predicts for a poor response/prognosis in 2 ER+ patient cohorts. Targeting of NOTCH4 reverses the increase in Notch and BCSC activity induced by anti-estrogens. Importantly, in PDX tumors with acquired tamoxifen resistance, NOTCH4 inhibition reduced BCSC activity. Thus, we establish that BCSC and NOTCH4 activities predict both de novo and acquired tamoxifen resistance and that combining endocrine therapy with targeting JAG1-NOTCH4 overcomes resistance in human breast cancers.

## Highlights

• Anti-estrogen therapies selectively enrich for BCSCs and activate Notch signaling
• Notch pathway activation and ALDH1 predict for anti-estrogen treatment failure
• Targeting of Notch4 reduces the population of BCSCs
• Notch inhibitors might prevent relapse or overcome resistance in ER+ tumors

## Graphical Abstract

**Figure:**
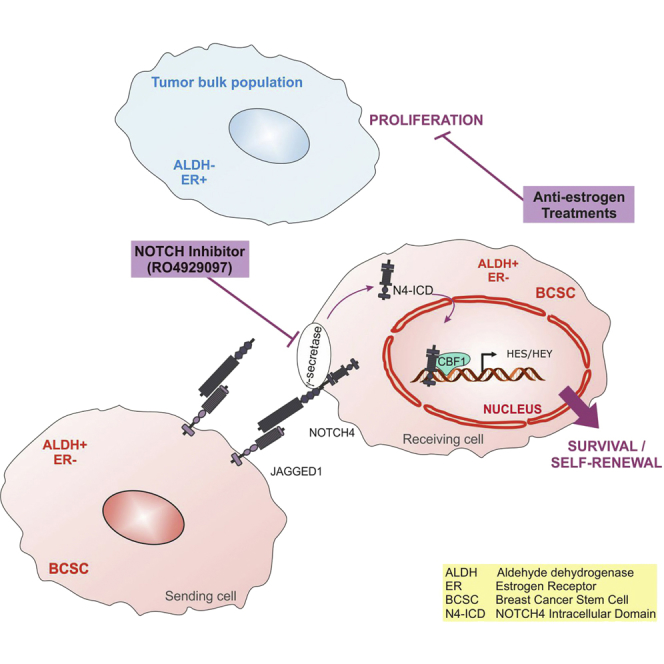

## Teaser

Breast cancers frequently develop resistance to anti-estrogen treatment, which makes it imperative to understanding how therapy resistance develops. Here, Simões et al. show that combining standard anti-estrogen therapies with anti-Notch4 drugs targeting breast cancer stem cells should improve treatment of ER+ breast cancer patients by preventing relapse due to therapy resistance.

## Introduction

Resistance to endocrine therapies such as selective estrogen receptor (ER) modulators (SERMs; e.g., tamoxifen), selective ER downregulators (SERDs; e.g., fulvestrant), and the aromatase inhibitors is seen in 50%–60% of early breast cancer (BC) cases and develops in almost all patients with advanced disease (Davies et al., 2011; Palmieri et al., 2014).

Evidence suggests that tumor-initiating or cancer stem-like cells (CSCs) are responsible for tumor recurrence after chemo- and endocrine therapy (Li et al., 2008; Creighton et al., 2009). Al-Hajj et al. (2003) were the first to show that tumor-initiating cells were capable of recapitulating the original tumor phenotype when transplanted into immunodeficient mice. In vitro functional assays for BC stem cell (BCSC) activity include aldehyde dehydrogenase 1 (ALDH1) enzyme activity and the capacity to form clonogenic mammospheres in suspension culture (Ginestier et al., 2007). It has been demonstrated that the BCSC population is ER negative/low and resistant to the direct effects of endocrine therapy (Simões et al., 2011; Harrison et al., 2013; Piva et al., 2014).

We have shown that aberrant Notch activation transforms normal breast cells, is found in pre-invasive and invasive human BCs, and correlates with early recurrence (Stylianou et al., 2006; Farnie et al., 2007). Moreover, we reported that inhibition of Notch signaling, particularly NOTCH4 receptor, reduced BCSC activity (Harrison et al., 2010).

Here, using patient-derived ER+ BC samples and patient-derived xenografts (PDXs), we report that short-term treatment with endocrine therapies enriches for JAG1-NOTCH4-regulated BCSCs, suggesting that these effects are not through genetic selection. Furthermore, we show that ALDH1 expression and NOTCH4 activation in human primary tumors are predictive of resistance to endocrine treatments. Finally, we demonstrate that NOTCH inhibition in vivo reduces BCSC activity in long-term acquired resistant PDX tumors. Thus, we propose that inhibiting Notch signaling will help overcome endocrine therapy resistance and recurrence in ER+ BC.

## Results

### BCSC Activity Is Enriched by Tamoxifen and Fulvestrant

**Figure 1:**
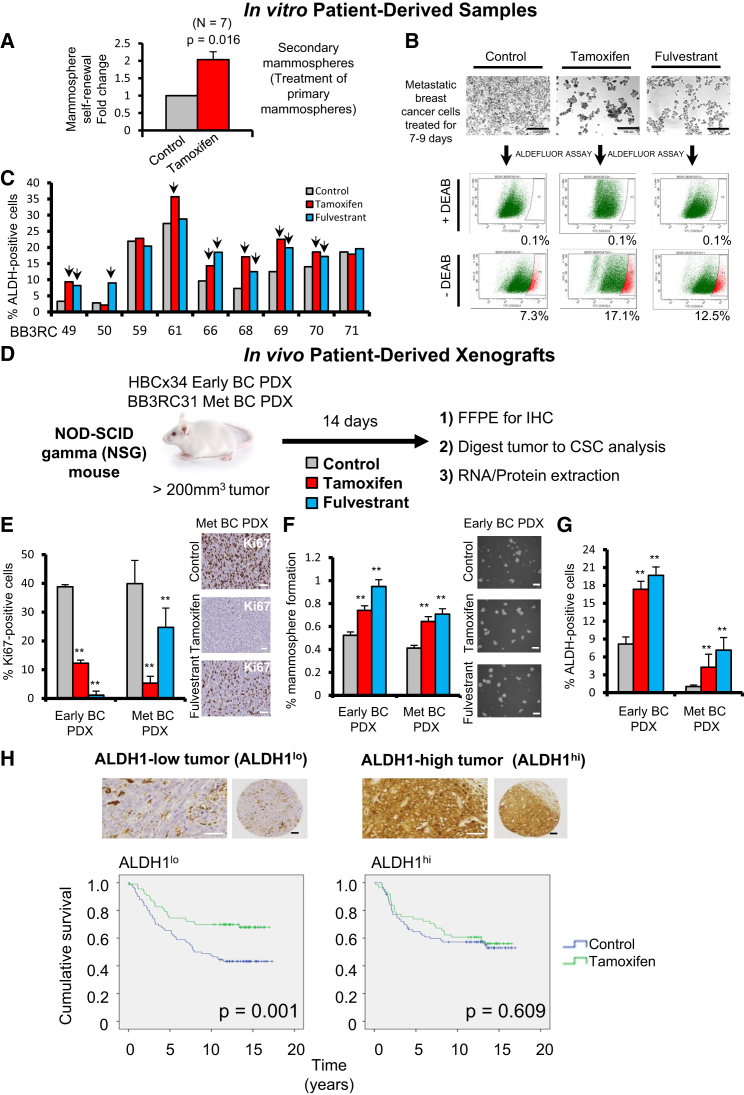
Tamoxifen or Fulvestrant Treatment of ER+ Patient-Derived Samples and PDXs Selectively Enriches for Cells with CSC Properties High BCSC frequency is associated with worse outcomes for tamoxifen-treated BC patients. (A) Mammosphere self-renewal of freshly isolated ER+ early and metastatic patient-derived samples. Primary mammospheres cultured in the presence of ethanol (Control) or 10^−6^ M 4-hydroxy-tamoxifen (Tamoxifen) were dissociated and re-plated in secondary mammosphere suspension culture for a further 7–9 days to measure self-renewal of mammosphere-initiating cells treated in the first generation. p value was calculated with Wilcoxon signed-rank test. (B) Representative micrographs of metastatic BC cells before fluorescence-activated cell sorting (FACS) analysis of ALDH1 enzymatic activity (ALDEFLUOR assay). ALDH-positive cells were discriminated from ALDH-negative cells using the ALDH inhibitor DEAB. (C) Percentage of ALDH-positive cells in nine ER+ metastatic BC patient-derived samples. Cells were grown in adherence with ethanol (Control), tamoxifen (10^−6^ M), or fulvestrant (10^−7^ M) for 7–9 days. Arrows indicate fold change greater than 20% compared to control. (D–G) Early (HBCx34) and metastatic (BB3RC31) BC estrogen-dependent PDX tumors treated in vivo for 14 days with tamoxifen (10 mg/kg/day, oral gavage; red bars) or fulvestrant (200 mg/kg/week, subcutaneous injection; blue bars). Gray bars correspond to vehicle control. FFPE, formalin-fixed paraffin-embedded. (E) Representative micrographs and quantification of Ki67 expression determined by immunohistochemistry (IHC). (F) Percentage of MFE. (G) ALDH-positive cells (%) determined using the ALDEFLUOR assay. (H) ALDH1 expression was assessed by immunohistochemistry in breast tumor epithelial cells, and the percentage of positive cells was scored. Representative micrographs of ALDH-high (ALDH^hi^) and -low (ALDH^lo^) epithelial expression are shown. Kaplan-Meier curves represent cumulative survival for the ALDH^lo^ population and ALDH^hi^ population of a cohort of 322 pre-menopausal ER+ BC patients who participated in a randomized trial of 2 years of adjuvant tamoxifen treatment versus no systemic treatment (control). Vertical bars on survival curves indicate censored cases. p values are based on a log-rank (Mantel-Cox) test of equality of survival distributions. Scale bars, 100 μm. Data are represented as mean ± SEM. ^∗^p < 0.05; ^∗∗^p < 0.01. See also Figure S1.

We tested the effect of the anti-estrogen tamoxifen on the mammosphere-forming efficiency (MFE) of patient-derived ER+ tumor cells and found that tamoxifen increases mammosphere self-renewal by about 2-fold (Figures 1A, S1A, and S1B). Next, we investigated ALDH activity, another functional assay for CSCs, in nine patient samples treated with tamoxifen or fulvestrant and showed significant increases in ALDH enzymatic activity in seven patients (Figures 1B and 1C). These data suggest that endocrine therapies, given for a period of a few days, enrich for stem cell activity.

Then, we tested the in vivo impact of endocrine therapies on stem cell activity in ER+ BC using PDXs grown subcutaneously in mice. We used both an early (treatment-naive; early BC) and a metastatic ER+ PDX tumor that both maintain biological characteristics (such as the expression of ER and estrogen dependence) of the patient primary tumor from which they were derived (Figures S1D and S1E). The estrogen dependence of the HBCx34 PDX model (early BC) has been previously reported (Cottu et al., 2012). Using a 14-day in vivo “window” treatment (Figure 1D), we showed that both tamoxifen and fulvestrant treatment decrease proliferation (Figure 1E). However, there is an increase in MFE and ALDH enzymatic activity (Figures 1F and 1G), suggesting a mechanism for endocrine resistance driven by enrichment for a stem cell phenotype.

The mechanism for this enrichment by anti-estrogens may be partly explained by more than 90% of sorted ALDH-positive cells being ER negative (Figure S1C). Thus, we hypothesized that frequency of ALDH-positive cells would predict for response to tamoxifen treatment, and we analyzed ALDH1 in 322 ER+ BC samples taken prior to a randomized trial of tamoxifen versus no systemic treatment. ALDH1 percentage dichotomized at the median value predicted benefit from tamoxifen so that improvement in survival (i.e., a response to treatment) was only seen in women with low epithelial ALDH1 expression (Figure 1H; Table S3). We saw no significant difference in recurrence between control treated patients with high versus low ALDH1 expression (p = 0.59). These data, from a prospective randomized trial, establish for the first time that ALDH-positive cell frequency predicts response to tamoxifen treatment, suggesting that stem cell numbers may be responsible for de novo endocrine resistance.

### Tamoxifen or Fulvestrant Treatment Upregulates Notch Target Genes

**Figure 2:**
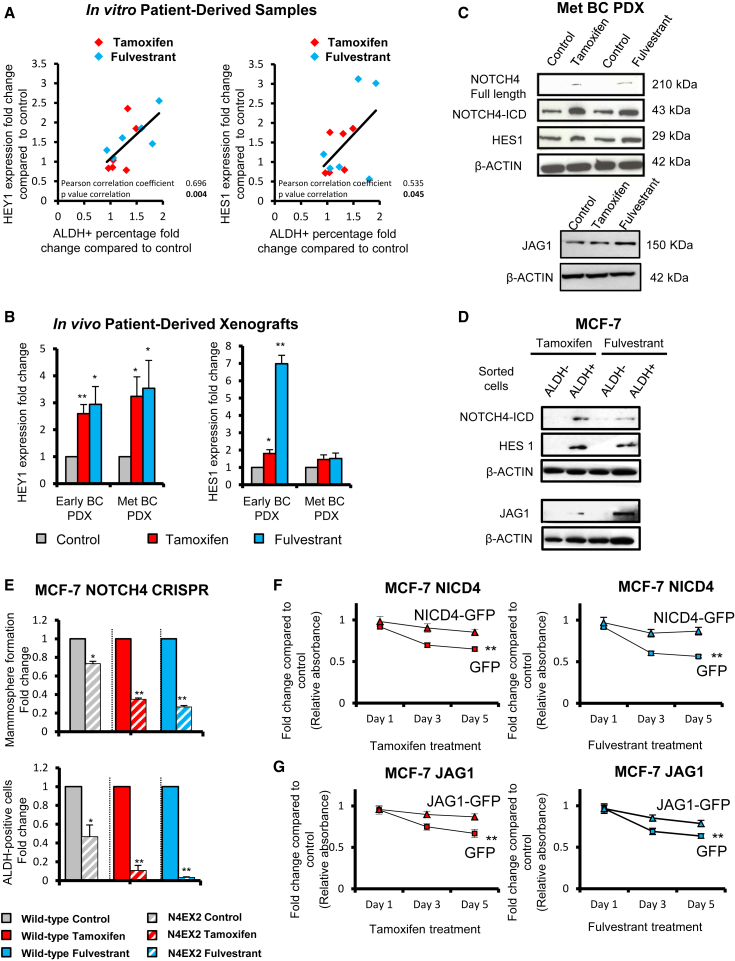
Tamoxifen or Fulvestrant Treatment Upregulates Notch Target Genes in Patient-Derived Samples and PDXs JAG1-NOTCH4 receptor signaling in ALDH-positive cells drives Notch activity in endocrine-resistant BC. (A and B) Expression of Notch target genes *HEY1* and *HES1* was assessed by real-time qPCR analysis and compared to control to determine fold change. (A) Metastatic BC patient-derived cells were treated for 7–9 days with ethanol (control), tamoxifen (10^−6^ M), or fulvestrant (10^−7^ M) and a correlation between fold change of expression of HEY1 and HES1 and fold change of percentage of ALDH-positive cells is shown. (B) Early (HBCx34) and metastatic (BB3RC31) BC PDXs: the effect of in vivo treatment for 14 days with tamoxifen (10 mg/kg/day, oral gavage) or fulvestrant (200 mg/kg/week, subcutaneous injection) on *HEY1* and *HES1*. (C) NOTCH4, HES1, and JAG1 protein expression levels determined by western blot in metastatic (Met) (BB3RC31) BC PDX. β-actin was used as a reference for the loading control. (D) NOTCH4, HES1, and JAG1 protein expression levels were determined by western blot in MCF-7 ALDH-negative and ALDH-positive sorted cells. MCF-7 cells were treated with tamoxifen or fulvestrant for 6 days before ALDH sorting. (E) Wild-type MCF-7 cells (filled bars) and a CRISPR clone containing a disruption of *NOTCH4* exon 2 (N4EX2 cells, hatched bars) treated in adherence with ethanol (Control, gray bars), 10^−6^ M tamoxifen (red bars), and 10^−7^ M fulvestrant (blue bars) for 6 days. N4EX2 cells’ fold change of MFE and ALDH-positive cells after treatments was compared to that of the wild-type cells. (F and G) NICD4 and JAG1 rescue tamoxifen- or fulvestrant-inhibited growth: cell number (using sulforhodamine B [SRB] assay, y axis) of MCF-7 overexpressing (F) NICD4-GFP, (G) JAG1-GFP, or GFP control incubated with tamoxifen or fulvestrant for 1, 3, and 5 days (x axis) compared to the respective cell line treated with control ethanol. p values are for the 5-day treatment. Data are represented as mean ± SEM. ^∗^p < 0.05; ^∗∗^p < 0.01. See also Figures S2 and S4.

We analyzed the patient-derived BC cells that were treated with tamoxifen and fulvestrant in Figures 1B and 1C and found that increased numbers of ALDH-positive cells were strongly correlated to increased expression of Notch target genes (*HEY1* and *HES1*) (Figure 2A). In addition, the BC PDX tumors treated in vivo with tamoxifen or fulvestrant (Figure 1D) for 2 weeks showed increased *HEY1* and *HES1* expression (Figure 2B), supporting an increased role for the Notch signaling pathway after endocrine therapies.

In ER+ cell lines (MCF-7, T47D, and ZR-75-1) in vitro, treatment with tamoxifen or fulvestrant for 6 days preferentially increased expression of *HEY1* and *HES1* (Figure S2A). Similarly, in tamoxifen-resistant (TAMR) or fulvestrant-resistant (FULVR) MCF-7 models, which have acquired resistance after long-term tamoxifen or fulvestrant treatment, we found upregulation of Notch target genes and increased Notch transcriptional activity (Figure S4A).

### JAG1 and NOTCH4 Receptor Signaling Drives Endocrine Resistance

Next, we assessed the expression of Notch receptors and ligands in parental, TAMR, and FULVR cell lines. NOTCH4 and its intracellular domain (ICD) were upregulated while NOTCH1, -2, and -3 were downregulated (Figure S4B) in the resistant versus parental cell lines. We found the Notch ligand JAG1 to be highly expressed in both resistant models (Figure S4B), while expression of the other four ligands was either unchanged (DLL1 and DLL4; Figure S4B) or absent (JAG2 and DLL3; data not shown). JAG1 and NOTCH4-ICD were also upregulated after a 14-day window treatment of PDXs in vivo, and after short-term treatment with tamoxifen or fulvestrant of MCF-7 cells in vitro, suggesting that activation of Notch signaling (demonstrated by increased HES1 expression) is an early event in the acquisition of endocrine resistance (Figures 2C and S2B). Importantly, JAG1, NOTCH4-ICD, and HES1 are expressed at higher levels in ALDH-positive cells, which suggests JAG1-NOTCH4 signaling between ALDH-positive cells (Figure 2D).

To further confirm the role of NOTCH4 activity in endocrine resistance and the stem cell phenotype, we analyzed loss-of- and gain-of-function phenotypes for NOTCH4-ICD in MCF-7 cells. Genomic disruption of exon 2 of *NOTCH4* by using a CRISPR approach led to loss of protein expression (Figures S2C–S2E) and a significant inhibition of MFE and ALDH-positive cells, especially after tamoxifen and fulvestrant treatments (Figure 2E). In contrast, overexpression of NOTCH4-ICD or JAG1 conferred tamoxifen and fulvestrant resistance in parental MCF-7 cells (Figures 2F and 2G).

Overall, these results indicate that JAG1 ligand and cleavage of NOTCH4-ICD may be responsible for Notch signaling activation after endocrine treatment, which is in agreement with recent reports that NOTCH4 expression is increased in TAMR cell lines (Yun et al., 2013; Lombardo et al., 2014).

### GSI RO4929097 Abrogates Tamoxifen- and Fulvestrant-Stimulated CSC Activity

**Figure 3:**
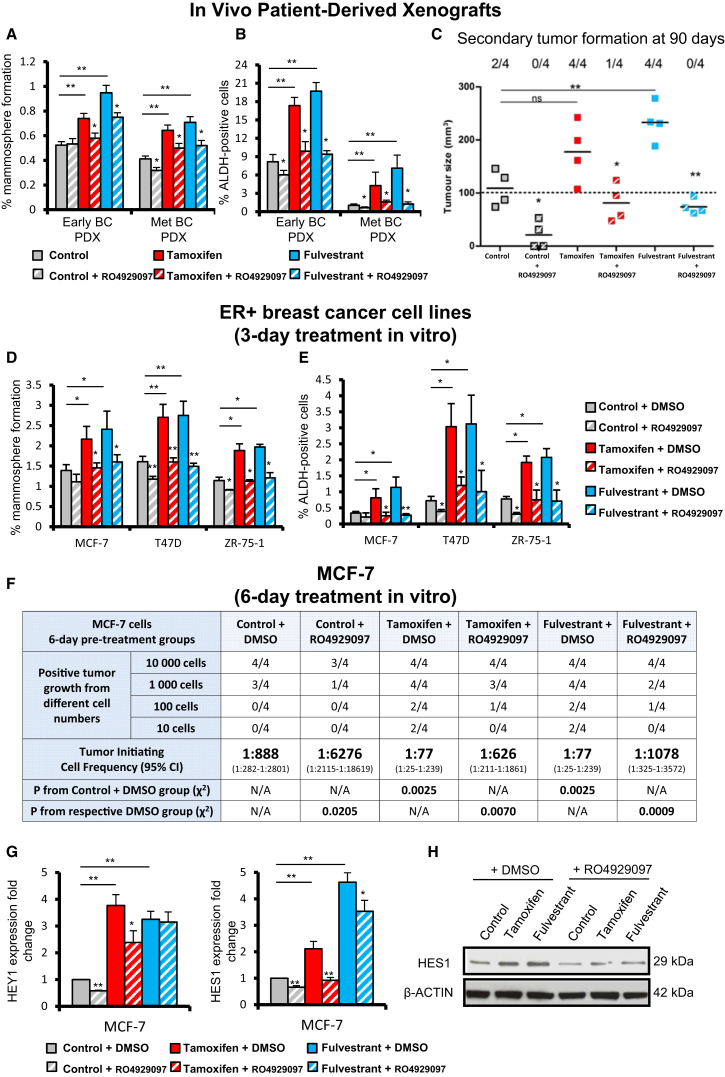
NOTCH4 Inhibition using RO4929097 Abrogates Tamoxifen and Fulvestrant Enrichment of CSC Activities (A–C) Early (HBCx34) and metastatic (Met) (BB3RC31) PDX tumors treated in vivo for 14 days with tamoxifen (10 mg/kg/day, oral gavage) or fulvestrant (200 mg/kg/week, subcutaneous injection) in the presence or absence of the NOTCH4 inhibitor RO4929097 (3 mg/kg/day, oral gavage). (A) MFE (%). (B) Percentage of ALDH-positive cells. (C) Secondary tumor formation. 100,000 cells of metastatic (BB3RC31) PDX were re-implanted subcutaneously in NSG mice with 90-day slow-release estrogen pellets. Tumor growth (>100 mm^3^) was determined at day 90 after cell injection. (D and E) MCF-7, T47D, and ZR-75-1 cells were pre-treated in adherence with 10^−6^ M tamoxifen (red bars) and 10^−7^ M fulvestrant (blue bars) with RO4929097 (10 μM; hatched bars) or DMSO (filled bars) for 72 hr. (D) MFE and (E) percentage of ALDH-positive cells were assessed after pre-treatments. (F–H) MCF-7 cells were pre-treated in adherence for 6 days in the presence of RO4929097 (10 μM; hatched bars) or DMSO (filled bars). (F) In vivo experiments were carried out in NSG mice with 90-day slow-release estrogen pellets. Tumor growth (>100 mm^3^) was assessed at day 60 and is represented as mice positive for growth/mice tested for each cell number tested. ELDA of tumor-initiating cell frequency is shown. (G) Expression of *HEY1* and *HES1* by real-time PCR was compared to the control. (H) HES1 protein expression levels determined by western blot. Data are represented as mean ± SEM. p values refer to hatched bars compared to filled control bars. ^∗^p < 0.05; ^∗∗^p < 0.01. See also Figures S3 and S4.

In order to inhibit NOTCH4 signaling, we used the gamma-secretase inhibitor (GSI) RO4929097, which we found to be effective in reducing levels of the active NOTCH4 ICD in endocrine-resistant models (Figure S4C). RO4929097 inhibited *HEY1* and *HES1* expression, as well as CBF1-Notch transcriptional activity in TAMR and FULVR cell lines, but not in parental MCF-7 cells (Figure S4D). Therefore, we tested whether RO4929097 would abrogate increases in MFE and ALDH-positive cells induced in vivo by anti-estrogens administered in short-term window treatments, using the same estrogen-dependent ER+ PDX tumors as in Figures 1D–1G. Tamoxifen and fulvestrant treatments reduced tumor growth and proliferation (Figures S3A and S3B) while increasing both MFE and ALDH activity (Figures 3A and 3B). RO4929097 had no impact on growth or proliferation (% Ki67; Figure S3B) but significantly inhibited endocrine-stimulated MFE and ALDH activity (Figures 3A and 3B). The gold standard for functionally determining tumor-initiating cells is xenograft formation in secondary mouse hosts, which we performed using dissociated cells from PDX tumors treated in vivo with anti-estrogens and/or RO4929097. Cells isolated from tumors treated in vivo with RO4929097 had significantly reduced tumor-initiating capacity 90 days post-implantation (Figure 3C). Furthermore, the stimulation of tumorigenicity following in vivo tamoxifen and fulvestrant treatment was completely reversed by RO4929097 (Figure 3C). Overall, these data suggest that BCSCs, measured by tumor-initiating activity and enriched by short-term anti-estrogen treatments, are dependent on NOTCH4 signaling that can be blocked by combination treatment with a NOTCH4 inhibitor.

To further substantiate this finding, we analyzed MFE and ALDH activity of MCF-7, T47D, and ZR-75-1 cells treated for 3 days with tamoxifen or fulvestrant in combination with RO4929097. In all cases, RO4929097 reduced MFE and ALDH-positive cells (Figures 3D and 3E). To confirm that RO4929097 reduced the tumor-initiating capacity, we conducted in vivo limiting dilution transplantation of MCF-7 cells. Extreme limiting dilution analysis (ELDA) revealed an 11-fold enrichment in tumor-initiating cell frequency following tamoxifen or fulvestrant pre-treatment, which was reversed by co-treatment with RO4929097 (Figure 3F). Inhibition of NOTCH4 cleavage/activation by RO4929097 was evidenced by decreased HEY1 and HES1 mRNA and protein levels (Figures 3G and 3H). Thus, we established, using PDX models and cell lines in tumor-initiating cell assays, that NOTCH4 inhibition reduces BCSC activity induced by anti-estrogen treatment.

### NOTCH4 Inhibition Targets CSCs in TAMR PDX Models

**Figure 4:**
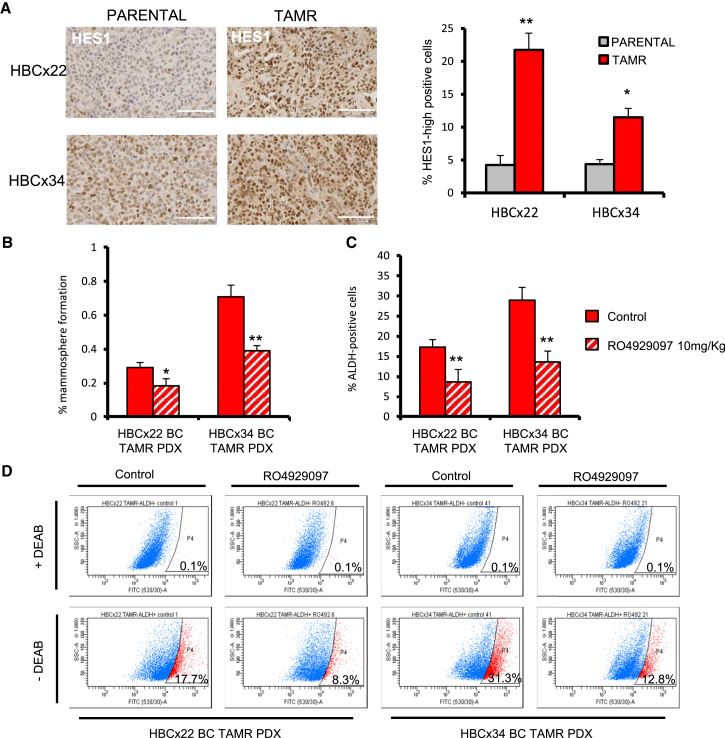
HBCx22 and HBCx34 TAMR PDXs Express High Levels of HES1 NOTCH4 inhibitor RO4929097 targets CSCs in tamoxifen-resistant (TAMR) PDXs. (A) Representative micrographs and quantification of HES1 expression determined by immunohistochemistry. Scale bars, 100 μm. (B–D) HBCx22 and HBCx34 TAMR PDXs treated in vivo for 14 days in the presence or absence of the GSI RO4929097 (10 mg/kg/day, oral gavage). (B) MFE (%). (C) Percentage of ALDH-positive cells. (D) Representative FACS plots of ALDEFLUOR assay. ALDH-positive cells were discriminated from ALDH-negative cells using the ALDH inhibitor DEAB. Data are represented as mean ± SEM. ^∗^p < 0.05; ^∗∗^p < 0.01. See also Figure S4.

The next question we asked was whether inhibiting NOTCH4 signaling to target BCSCs will overcome long-term acquired anti-estrogen resistance in ER+ BC patients. We investigated RO4929097 treatment in two established PDXs (HBCx22 and HBCx34) that have long-term acquired resistance to tamoxifen in vivo. Analysis of HES1 expression by immunohistochemistry revealed that these two TAMR PDXs displayed increased Notch signaling activation compared to the parental control (Figure 4A). Notably, the TAMR HBCx34 PDX model has a higher percentage of MFE and ALDH activity than the endocrine-sensitive HBCx34 PDX model (compare Figures 1F and 1G with Figures 4B and 4C). These data suggest that acquired tamoxifen resistance in PDX models involves enrichment for BCSC activity through Notch signaling. Treatment with RO4929097 for 14 days demonstrates that MFE and ALDH activity can be significantly reduced in TAMR PDX tumors in vivo (Figures 4B–4D).

### NOTCH4/HES/HEY Gene Signature Predicts for Resistance to Tamoxifen Treatment and Prognosis in ER+ Tumors

**Figure 5:**
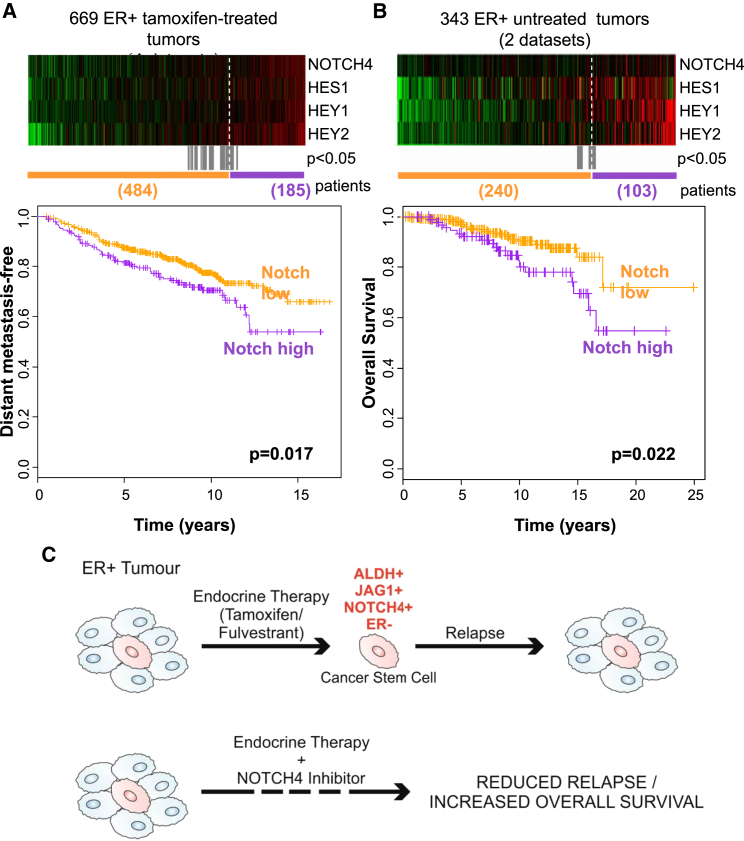
NOTCH4 Receptor Activity Predicts for Resistance to Tamoxifen Treatment and Prognosis in ER+ Tumors (A and B) *NOTCH4*, *HES1*, *HEY1*, and *HEY2* genes in ER+ primary tumors from (A) tamoxifen-treated or (B) untreated patients are co-expressed in the heatmap ranked from left to right using the four-gene signature. Colors are log_2_ mean-centered values; red indicates high, and green indicates low. All significant cut-points (p < 0.05) are shown in gray. Kaplan-Meier analysis using the optimum cut-point (dashed white line) demonstrates that elevated expression of the Notch genes is significantly associated with an increased rate of (A) distant metastasis and (B) decreased overall survival. Vertical bars on survival curves indicate censored cases. p values are based on a log-rank (Mantel-Cox) test. (C) Diagram suggesting that endocrine therapies do not target BCSCs and emphasizing the need of targeting residual drug-resistant cells to eliminate all cancer cells and prevent long-term recurrences of ER+ BC.

Based on the aforementioned observations, we hypothesized that NOTCH4 activity, comprising a NOTCH4/HES/HEY gene signature, would predict for response to tamoxifen treatment. In gene expression data from 669 pre-treatment tumors from four published Affymetrix microarray datasets of ER+ patients who subsequently received adjuvant tamoxifen therapy, we found *NOTCH4*, *HES1*, *HEY1*, and *HEY2* to be co-expressed in some tumors, as demonstrated in the heatmap ordered from left to right by the sum of the four genes (Figure 5A). Importantly, elevated expression of these Notch genes before treatment was significantly associated with distant metastasis (Figure 5A) and with reduced overall survival in an independent cohort of 343 untreated ER+ patients (Figure 5B). Thus, *NOTCH4* gene expression and activity in tumors before treatment with endocrine therapy predicts sensitivity to treatment, indicating that this signaling pathway predicts de novo as well as acquired endocrine resistance. These data strengthen the case for therapies against NOTCH4 to target the endocrine-resistant ALDH-positive cells responsible for relapse of ER+ tumors following hormonal therapy (Figure 5C).

## Discussion

Here, we report that BCSC activity and frequency are increased in response to the common endocrine therapies tamoxifen and fulvestrant in ER+ patient samples and in early and metastatic PDXs. Our findings suggest that endocrine therapies do not target BCSCs, and this may explain how residual drug-resistant cells are responsible for the relapse of ER+ tumors following hormonal therapy. Although we observe increased BCSC frequency after endocrine treatments, we do not know whether absolute BCSC numbers remain the same and are selected for or whether they can be induced by anti-estrogen treatment. Tamoxifen and fulvestrant are clearly successful in reducing BC recurrence in some patients. In other patients with poorer outcome after endocrine therapies, we demonstrate that tumors have high pre-treatment levels of ALDH1 expression and NOTCH4 activation. Moreover, we found that treating ER+ BC cells with endocrine therapies specifically increases JAG1-NOTCH4 signaling and that combining endocrine therapies with a Notch pathway inhibitor can prevent BCSC enrichment induced by endocrine therapies. Thus, our findings in patient-derived BCSCs establish that JAG1 ligand signaling through the NOTCH4 receptor in ALDH-positive cell populations is a determining factor in the acquisition of endocrine resistance.

The best described strategy for inhibition of Notch signaling is the use of small-molecule GSIs, which prevent the release of Notch ICD (NICD). In our study, the GSI RO4929097 specifically targets NOTCH4 cleavage in anti-estrogen-treated cells and, thus, decreases BCSC activity in vitro (MFE and ALDH activity) and tumor initiation in vivo. Our investigations in ER+ PDX tumors provide the rationale for the use of NOTCH4 inhibitors together with endocrine therapies in the adjuvant or advanced settings (Figure 5C). Significantly, we demonstrated the utility of RO4929097 to target BCSCs in pre-clinical models of TAMR patient tumors.

In conclusion, our data establish that tamoxifen and fulvestrant select for stem cell activity in short- and long-term-treated BC cells, as well as in early endocrine therapy naive and metastatic-endocrine-treated patient-derived samples and PDXs. Importantly, we report that low numbers of stem cells and low Notch signaling activation in patient tumors predict response to tamoxifen therapy and better survival. Overall, these results suggest that ER+ BC recurrence after endocrine therapies, which target the majority of cells (ER+ cells), will be reduced by targeting the JAG1+/NOTCH4+/ALDH1+/ER− BCSC population.

## Experimental Procedures

### Patient-Derived Samples

Early BC samples were collected in RPMI (GIBCO), dissected into 1- to 2-mm^3^ cubes and digested with the Human Tumor Dissociation Kit (Miltenyi Biotec) for 2 hr at 37°C. Digested tissue was filtered sequentially through 100- and 40-μm cell strainers, then centrifuged at 300 × g for 5 min and washed in PBS.

Metastatic samples (ascites or pleural effusions) were centrifuged at 1,000 × g for 10 min at 4°C. The cell pellets were diluted in PBS. Erythrocytes and leucocytes were removed using Lymphoprep (Axis-Shield) and CD45-negative magnetic sorting (Miltenyi Biotec), respectively. Cells were cultured in adherence for 7–9 days in DMEM/F-12 medium, GlutaMAX (GIBCO) with 10% fetal bovine serum (FBS; GIBCO), 10 μg/ml insulin (Sigma-Aldrich), 10 μg/ml hydrocortisone (Sigma-Aldrich), and 5 ng/ml epidermal growth factor (EGF; Sigma-Aldrich), in 10^−6^ M 4-OH tamoxifen (Sigma-Aldrich, H7904), 10^−7^ M fulvestrant (ICI 182,780, Tocris, 1047), or ethanol (control).

Clinico-pathological details of the samples are summarized in Tables S1 (primary BC) and S2 (metastatic BC).

Please refer to the Supplemental Experimental Procedures for further details.

### PDXs and In Vivo Experiments

Mouse studies commenced in 8- to 12-week-old female mice and were conducted in accordance with the UK Home Office Animals (Scientific Procedures) Act 1986, using NSG (NOD.Cg-*Prkdc*^*scid*^ *Il2rg*^*tm1Wjl*^/SzJ) mice. All in vivo work was performed with a minimum of n = 4 mice per condition.

Serial passaging of the PDX was carried out by implanting small fragments of the tumor subcutaneously into dorsal flanks of NSG mice. Early (HBCx34) and metastatic (BB3RC31) BC estrogen-dependent PDXs were administered with 8 μg/ml of 17-beta estradiol in drinking water at all times and were treated with drugs when tumors reached 200–300 mm^3^. Experiments were performed using PDX tumors between passages 5 and 8. Animal weight and tumor size was measured bi-dimensionally using callipers twice a week.

Tamoxifen citrate (Sigma, T9262, 10 mg/kg/day) and RO4929097 (Cellagen Technology, 3 mg/kg/day) were administered by oral gavage (0.1 ml per dose) on a basis of 5 days out of 7 (weekends excluded) for 14 days. Tamoxifen citrate and RO4929097 were prepared in 1% carboxymethylcellulose (Sigma, C9481) dissolved in distilled water. Fulvestrant (kindly provided by AstraZeneca, 200 mg/kg/week) was administered by subcutaneous injection (0.1 ml per dose) on a weekly basis for 14 days. The HBCx22 and HBCx34 TAMR PDXs were treated for 14 days in the presence or absence of the GSI RO4929097 (10 mg/kg/day, oral gavage). Xenografts were collected in ice-cold DMEM for live-cell assays, histological analysis, and RNA and protein extraction. PDX single-cell suspension was obtained using a collagenase-hyaluronidase mixture for digestion (Stem Cell Technologies).

Please refer to the Supplemental Experimental Procedures for further details.

### Mammosphere Colony Assay

MFE was calculated by dividing the number of mammospheres formed (≥50 μm) by the original number of single cells seeded (500 cells per square centimeter for primary cells) and is expressed as fold change normalized to control or as the mean percentage of MFE (Shaw et al., 2012).

Please refer to the Supplemental Experimental Procedures for further details.

### Tamoxifen Trial Study

Premenopausal BC patients with invasive stage II disease were enrolled in SBII:2a, a Swedish clinical trial in which patients were randomly assigned to receive 2 years of adjuvant tamoxifen or no treatment (control) and followed up for recurrence-free and overall survival (Rydén et al., 2005). Our data represent cumulative survival for a cohort of 322 premenopausal ER+ BC patients stratified by ALDH-low (below median) and ALDH-high (above median) expression over time.

### Notch Gene Expression Signature

The gene expression data on 669 ER+ tamoxifen-treated tumors (GSE6532, GSE9195, GSE17705, and GSE12093) and 343 ER+ untreated tumors (GSE2034 and GSE7390) are from published Affymetrix microarray datasets.

Please refer to the Supplemental Experimental Procedures for additional details.

### Statistical Analysis

If not stated otherwise, a two-tailed Student’s t test was performed for statistical analysis. A value of p < 0.05 was considered to be statistically significant. Error bars represent the SEM of at least three independent experiments. Data are shown as mean ± SEM.

## Author Contributions

B.M.S. and C.S.O.: conception and design, collection and/or assembly of data, data analysis and interpretation, and manuscript writing. R.E., A.S., L.Y., A.S.-C., D.G.A., K.S., A.S.-G., F.C., A.A., S.C., S.A., A.U., and G.F.: collection and/or assembly of data, data analysis and interpretation, and manuscript critique. A.G.: study recruitment, collection of BC tissue, and manuscript critique. A.H. and K.B.: conception, design, and manuscript critique. J.G.: generation of resistant cell lines and manuscript critique. L.R. and G.L.: Tamoxifen trial study and BC clinical pathology expertise and manuscript critique. A.H.S.: gene expression analysis and manuscript critique. E.M.: generation of PDX and manuscript critique. S.J.H. and R.B.C.: conception and design, data analysis and interpretation, manuscript writing, and final approval of manuscript.

## Supplemental Information

Document S1. Supplemental Experimental Procedures, Figures S1–S4, and Tables S1–S3

Document S2. Article plus Supplemental Information

## References

[bib1] Al-Hajj M., Wicha M.S., Benito-Hernandez A., Morrison S.J., Clarke M.F. (2003). Prospective identification of tumorigenic breast cancer cells. Proc. Natl. Acad. Sci. USA.

[bib2] Cottu P., Marangoni E., Assayag F., de Cremoux P., Vincent-Salomon A., Guyader Ch., de Plater L., Elbaz C., Karboul N., Fontaine J.J. (2012). Modeling of response to endocrine therapy in a panel of human luminal breast cancer xenografts. Breast Cancer Res. Treat..

[bib3] Creighton C.J., Li X., Landis M., Dixon J.M., Neumeister V.M., Sjolund A., Rimm D.L., Wong H., Rodriguez A., Herschkowitz J.I. (2009). Residual breast cancers after conventional therapy display mesenchymal as well as tumor-initiating features. Proc. Natl. Acad. Sci. USA.

[bib4] Davies C., Godwin J., Gray R., Clarke M., Cutter D., Darby S., McGale P., Pan H.C., Taylor C., Wang Y.C., Early Breast Cancer Trialists’ Collaborative Group (EBCTCG) (2011). Relevance of breast cancer hormone receptors and other factors to the efficacy of adjuvant tamoxifen: patient-level meta-analysis of randomised trials. Lancet.

[bib5] Farnie G., Clarke R.B., Spence K., Pinnock N., Brennan K., Anderson N.G., Bundred N.J. (2007). Novel cell culture technique for primary ductal carcinoma in situ: role of Notch and epidermal growth factor receptor signaling pathways. J. Natl. Cancer Inst..

[bib6] Ginestier C., Hur M.H., Charafe-Jauffret E., Monville F., Dutcher J., Brown M., Jacquemier J., Viens P., Kleer C.G., Liu S. (2007). ALDH1 is a marker of normal and malignant human mammary stem cells and a predictor of poor clinical outcome. Cell Stem Cell.

[bib7] Harrison H., Farnie G., Howell S.J., Rock R.E., Stylianou S., Brennan K.R., Bundred N.J., Clarke R.B. (2010). Regulation of breast cancer stem cell activity by signaling through the Notch4 receptor. Cancer Res..

[bib8] Harrison H., Simoes B.M., Rogerson L., Howell S.J., Landberg G., Clarke R.B. (2013). Oestrogen increases the activity of oestrogen receptor negative breast cancer stem cells through paracrine EGFR and Notch signalling. Breast Cancer Res..

[bib9] Li X., Lewis M.T., Huang J., Gutierrez C., Osborne C.K., Wu M.F., Hilsenbeck S.G., Pavlick A., Zhang X., Chamness G.C. (2008). Intrinsic resistance of tumorigenic breast cancer cells to chemotherapy. J. Natl. Cancer Inst..

[bib10] Lombardo Y., Faronato M., Filipovic A., Vircillo V., Magnani L., Coombes R.C. (2014). Nicastrin and Notch4 drive endocrine therapy resistance and epithelial to mesenchymal transition in MCF7 breast cancer cells. Breast Cancer Res..

[bib11] Palmieri C., Patten D.K., Januszewski A., Zucchini G., Howell S.J. (2014). Breast cancer: current and future endocrine therapies. Mol. Cell. Endocrinol..

[bib12] Piva M., Domenici G., Iriondo O., Rábano M., Simões B.M., Comaills V., Barredo I., López-Ruiz J.A., Zabalza I., Kypta R., Vivanco Md. (2014). Sox2 promotes tamoxifen resistance in breast cancer cells. EMBO Mol. Med..

[bib13] Rydén L., Jönsson P.E., Chebil G., Dufmats M., Fernö M., Jirström K., Källström A.C., Landberg G., Stål O., Thorstenson S. (2005). Two years of adjuvant tamoxifen in premenopausal patients with breast cancer: a randomised, controlled trial with long-term follow-up. Eur. J. Cancer.

[bib14] Shaw F.L., Harrison H., Spence K., Ablett M.P., Simões B.M., Farnie G., Clarke R.B. (2012). A detailed mammosphere assay protocol for the quantification of breast stem cell activity. J. Mammary Gland Biol. Neoplasia.

[bib15] Simões B.M., Piva M., Iriondo O., Comaills V., López-Ruiz J.A., Zabalza I., Mieza J.A., Acinas O., Vivanco M.D. (2011). Effects of estrogen on the proportion of stem cells in the breast. Breast Cancer Res. Treat..

[bib16] Stylianou S., Clarke R.B., Brennan K. (2006). Aberrant activation of notch signaling in human breast cancer. Cancer Res..

[bib17] Yun J., Pannuti A., Espinoza I., Zhu H., Hicks C., Zhu X., Caskey M., Rizzo P., D’Souza G., Backus K. (2013). Crosstalk between PKCα and Notch-4 in endocrine-resistant breast cancer cells. Oncogenesis.

