# Supplementary material for: Anti-estrogen Resistance in Human Breast Tumors Is Driven by JAG1-NOTCH4-Dependent Cancer Stem Cell Activity
Source: Cell Rep. 2015 Sep 17;12(12):1968–77. doi: 10.1016/j.celrep.2015.08.050 (PMC4594158; doi:10.1016/j.celrep.2015.08.050)
Supplement: Document S1. Supplemental Experimental Procedures, Figures S1–S4, and Tables S1–S3 [file mmc1.pdf]

Cell Reports

Supplemental Information

Anti-estrogen Resistance in Human Breast Tumors Is Driven by JAG1-NOTCH4-Dependent Cancer Stem Cell Activity

Bruno M. Simões, Ciara S. O'Brien, Rachel Eyre, Andreia Silva, Ling Yu, Aida Sarmiento-Castro, Denis G. Alférez, Kath Spence, Angélica Santiago-Gómez, Francesca Chemi, Ahmet Acar, Ashu Gandhi, Anthony Howell, Keith Brennan, Lisa Rydén, Stefania Catalano, Sebastiano Andó, Julia Gee, Ahmet Ucar, Andrew H. Sims, Elisabetta Marangoni, Gillian Farnie, Göran Landberg, Sacha J. Howell, and Robert B. Clarke

Supplementary figure 1

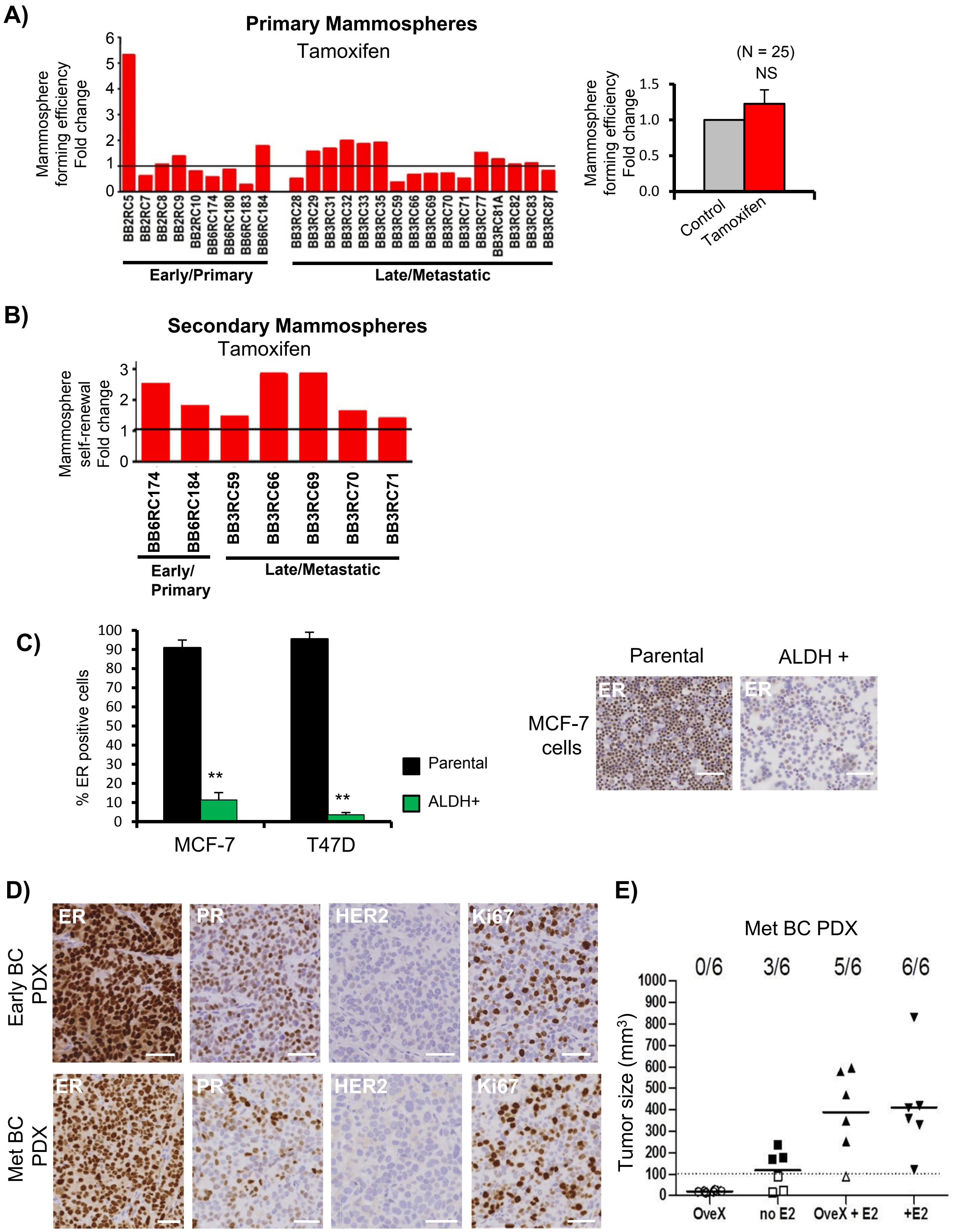

Supplementary figure 2

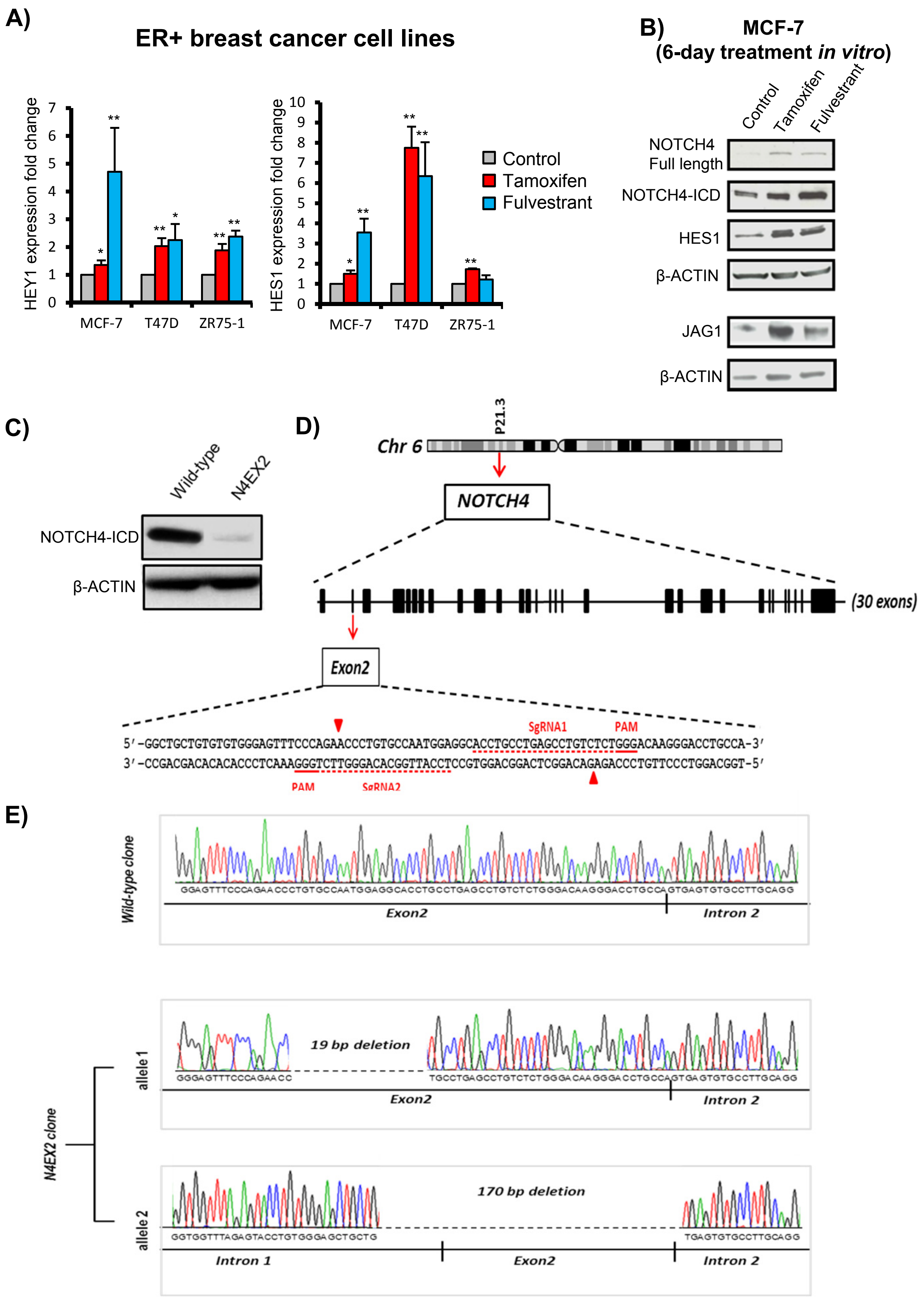

Supplementary figure 3

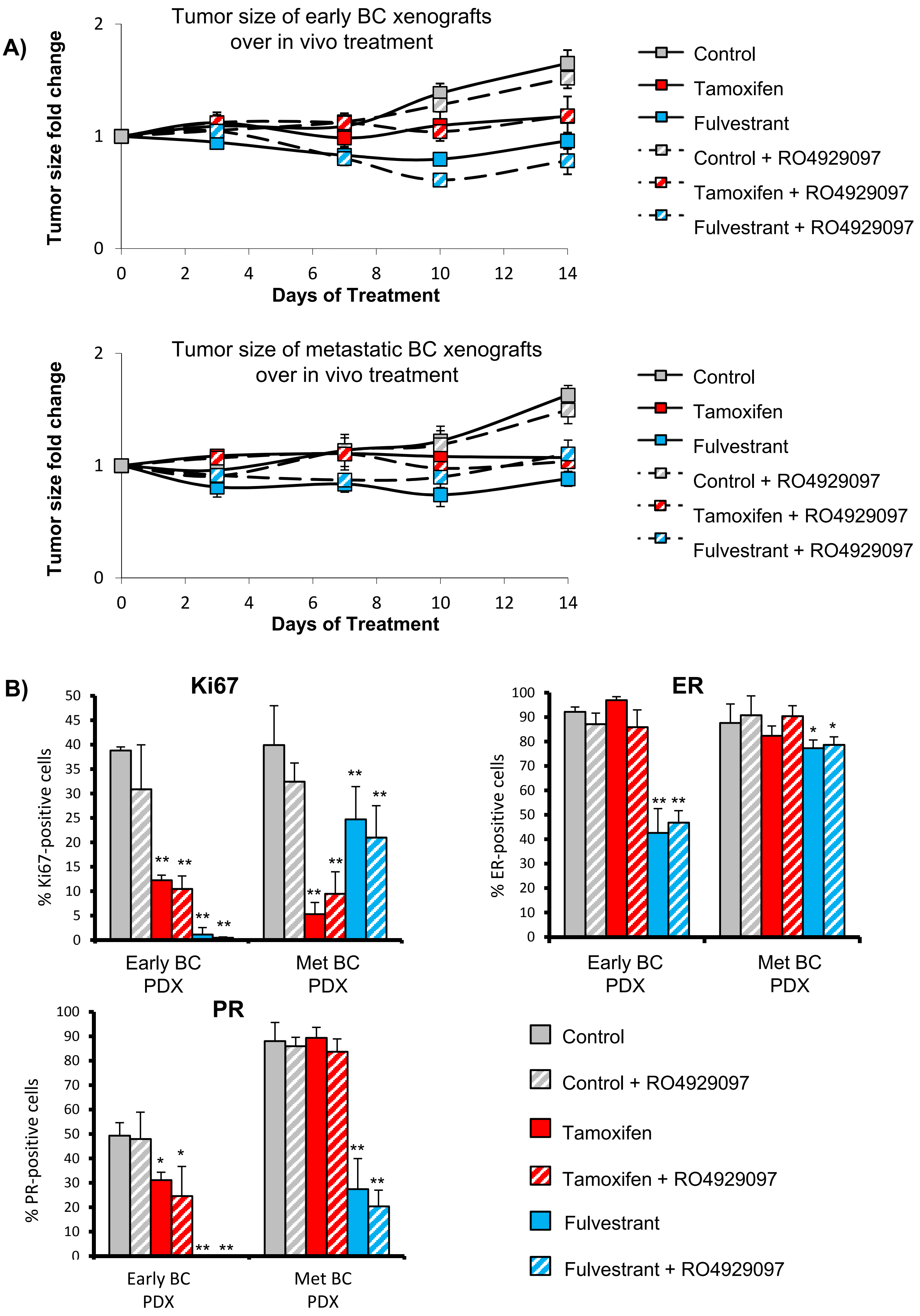

Supplementary figure 4

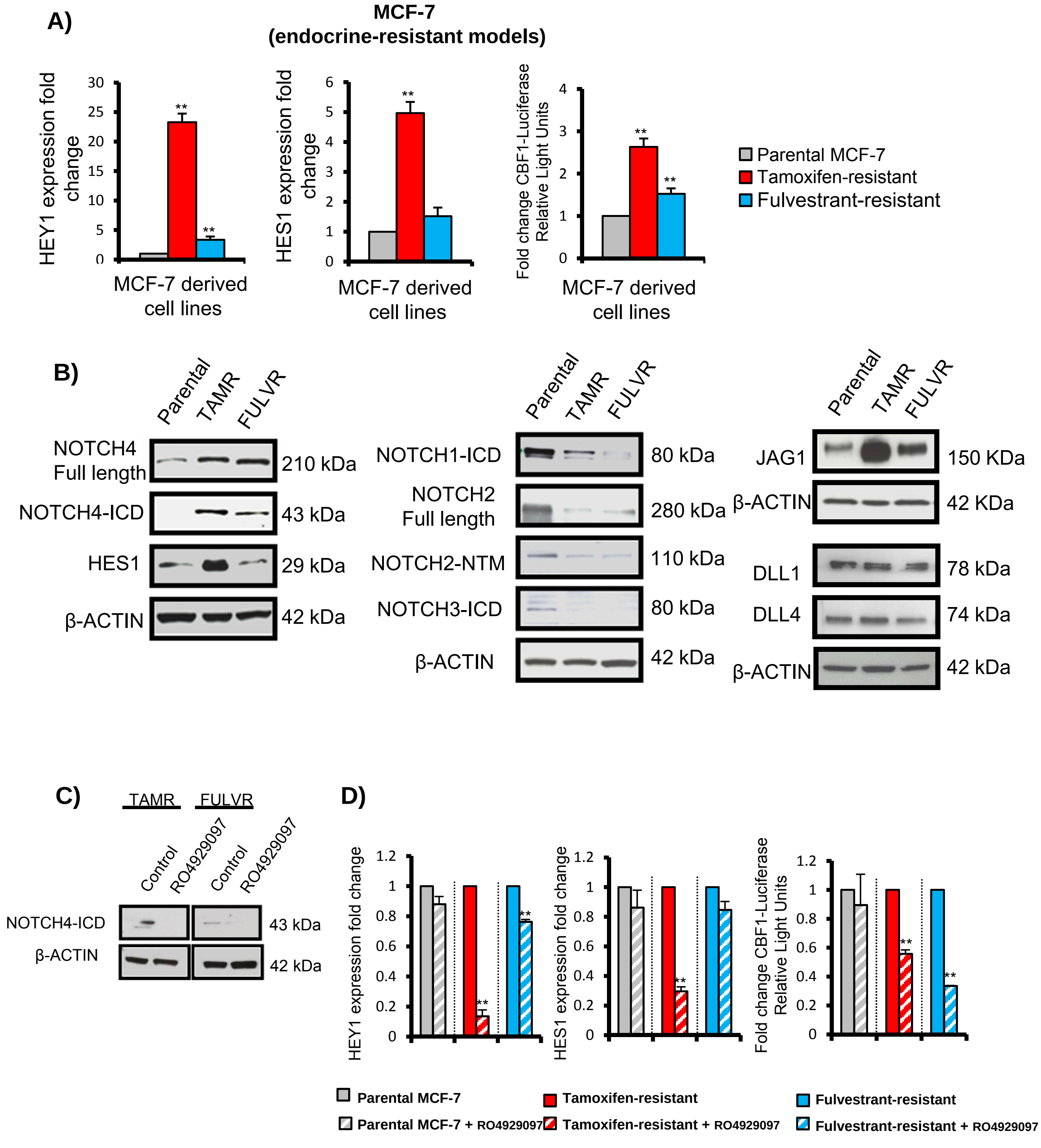

Supplemental Figure Legends

Figure S1, Related to Figure 1:

A) Mammosphere formation efficiency (MFE) of freshly isolated ER+ early and metastatic patient-derived samples cultured in the presence of ethanol (Control) or 10^{-6} M 4-hydroxy-tamoxifen. Mammosphere data for each individual patient sample is represented.

B) Primary mammospheres were dissociated and replated in secondary mammosphere suspension culture for a further 7-9 days to measure self-renewal of mammosphere-initiating cells treated in the first generation.

C) ER positive cells as a percentage of total cell population in parental and aldefluor positive (ALDH+) cells were determined in MCF7 and T47D cell lines by immunocytochemistry. The right hand panel shows representative micrographs of ER staining in MCF-7 cells. Data are represented as mean ± SEM. ** $p < 0.01$

D) Early (HBCx34) and metastatic (BB3RC31) xenograft tumor sections stained for ER, PR, HER2 and Ki67 by immunohistochemistry. Scale bars = 100µm.

E) Ovariectomized mice and administration of estrogen in the drinking water (8µg/ml) were used to perform an estrogen dependence test of metastatic BB3RC31 PDX. Graph shows tumor formation and size at 120 days after implantation. Tumor formation was determined by counting tumors greater than 100 mm3 (tumors bigger than 100 mm3 are represented by solid forms and tumors smaller than 100 mm3 are represented by hollow forms). Mean of each group is represented by horizontal bar. OveX- Ovariectomized; E2 – Estrogen

Figure S2, Related to Figure 2:

A) Expression of Notch target genes *HEY1* and *HES1* was assessed by quantitative real-time PCR analysis and compared to control to determine fold change. MCF-7, T47D and ZR-75-1 cells were treated with 10^{-6} M tamoxifen (red bar) and 10^{-7} M fulvestrant (blue bar) for six days. Data are represented as mean ± SEM. * $p < 0.05$, ** $p < 0.01$

B) NOTCH4, HES1 and JAG1 protein expression levels determined by Western Blot in MCF-7 cells treated with 10^{-6} M tamoxifen (red bar) and 10^{-7} M fulvestrant for 6 days. β-actin was used as a reference for the loading control.

C) NOTCH4 - intracellular domain (ICD) protein levels were determined in wild-type and N4EX2 CRISPR clone by Western Blot. NB: A very small amount of NOTCH4-ICD was detected even though N4EX2 clone contains a frameshift deletion in both alleles, which we hypothesize to be due to a small amount of in-frame RNA produced by whole exon 3 skipping, i.e., by splicing of exon 1 donor site to the acceptor sites of exons that are in-frame.

D) Schematic representation of *NOTCH4* location in chromosome 6 and sgRNA double-nickase CRISPR design to target exon 2 of *NOTCH4*. sgRNAs used are underlined in red dashed line. Predicted cleavage sites for single-strand break start 3 nucleotides upstream of the PAM sequence (underlined in red) and are indicated by the red arrows.

E) Sanger sequencing of wild-type and *NOTCH4* CRISPR targeted clone (N4EX2 clone) showing a deletion of 19 nucleotides in the exon 2 and a deletion of 170 nucleotides, which comprises the entire exon 2.

Figure S3, Related to Figure 3:

A) Early (HBCx34) and metastatic (BB3RC31) PDXs tumor size variation over 14 days in vivo treatments with tamoxifen (10mg/kg/day, oral gavage) or fulvestrant (200mg/kg/week, subcutaneous injection) in the presence or absence of the gamma-secretase inhibitor RO4929097 (3mg/kg/day, oral gavage). Tumor size was determined every 3-4 days and fold change was calculated by dividing the tumor size by the size of the respective tumor at day 0.

B) Quantification of Ki67, ER and PR expression determined by immunohistochemistry. Early (HBCx34) and metastatic (BB3RC31) PDX tumors treated in vivo for 14 days with tamoxifen (10mg/kg/day, oral gavage) or fulvestrant (200mg/kg/week, subcutaneous injection) in the presence or absence of the gamma-secretase inhibitor RO4929097 (3mg/kg/day, oral gavage). Data are represented as mean ± SEM. * $p < 0.05$; ** $p < 0.01$

Figure S4, Related to Figures 2, 3 and 4:

A) Expression of *HEY1* and *HES1* in endocrine-resistant cells compared to the parental MCF-7 cells (two left hand panels). Notch transcriptional activity in endocrine-resistant cells compared to control was determined by relative firefly luciferase activity of 10x CBF1 reporter (right hand panel).

B) Notch receptors and Notch ligands protein expression levels determined by Western Blot in MCF-7 endocrine-resistant cells. β-actin was used as a reference for the loading control.

C) NOTCH4 intracellular domain expression levels determined by Western Blot in MCF-7 endocrine-resistant cells treated with GSI RO4929097 or control DMSO for 72h.

D) Expression of *HEY1* and *HES1* and Notch transcriptional activity (10xCBF1-luciferase reporter) are inhibited by GSI RO4929097 (72h) in endocrine-resistant cells.

Data are represented as mean ± SEM. * $p < 0.05$, ** $p < 0.01$

TABLE S1: Characteristics of 'early' patient-derived tumors used in the study. PBC – primary breast cancer. (Related to Experimental Procedures)

	STAGE	TYPE	GRADE	ER	PgR	HER-2	Neoadjuvant Treatment
PBC 1 (BB2RC5)	Early	IDC	3	+	+	-	-
PBC 2 (BB2RC7)	Early	IDC	3	+	-	+	-
PBC 3 (BB2RC8)	Early	IDC	ND	+	-	-	-
PBC 4 (BB2RC9)	Early	IDC	3	+	+	+	-
PBC 5 (BB2RC10)	Early	IDC	2	+	+	-	Tamoxifen Letrozole
PBC 6 (BB6RC174)	Early	IDC	2	+	+	-	Tamoxifen
PBC 7 (BB6RC180)	Early	IDC	2	+	+	-	-
PBC 8 (BB6RC183)	Early	IDC/ILC	3	+	-	-	-
PBC 9 (BB6RC184)	Early	IDC	3	+	+	-	-

IDC: invasive ductal carcinoma; ILC: invasive lobular carcinoma; ND: not determined

TABLE S2: Characteristics of 'late' metastatic endocrine therapy-treated patient-derived tumors used in the study. Met BC – metastatic breast cancer. (Related to Experimental Procedures)

	TYPE	GRADE	ER	PgR	HER-2	Chemotherapy	Hormonal therapy	Targeted therapy
Met BC 1<br>(BB3RC28)	IDC	1	+	+	-	TOPIC 2 trial<br>Docetaxel Capecitabine	Tamoxifen<br>Exemestane<br>Fulvestrant	Sutent
Met BC 2<br>(BB3RC29)	ILC	2	+	+	-	FEC<br>Paclitaxel<br>Epirubicin	Anastrazole<br>Exemestane<br>Fulvestrant<br>Letrozole	
Met BC 3<br>(BB3RC31)	IDC	2	+	+	-	CMF	Tamoxifen<br>Letrozole<br>Exemestane<br>Low dose Estradiol	Gefitinib
Met BC 4<br>(BB3RC32)	IDC	2	+	+	-	FEC<br>Docetaxel<br>Paclitaxel	Anastrozole	
Met BC 5<br>(BB3RC33)	ILC	2	+	+	-	Epirubicin<br>Capecitabine	Tamoxifen<br>Exemestane<br>Letrozole	
Met BC 6<br>(BB3RC35)	IDC	2	+	+	-	Capecitabine<br>Docetaxel	Letrozole<br>Tamoxifen<br>Goserelin<br>Exemestane	
Met BC 7<br>(BB3RC49)	IDC	2	+	+	-	FEC<br>Paclitaxel	Letrozole<br>Tamoxifen<br>Exemestane	
Met BC 8<br>(BB3RC50)	IDC	2	+	+	-	FEC<br>Paclitaxel<br>Capecitabine<br>Vinorelbine	Tamoxifen<br>Anastrazole<br>Exemestane<br>Fulvestrant	
Met BC 9<br>(BB3RC59)<br>Met BC 9A<br>(BB3RC61)<br>Met BC 9B<br>(BB3RC66)	ILC	2	+	+	-	EOX<br>Capecitabine<br>Paclitaxel	Letrozole<br>Tamoxifen<br>Exemestane<br>Fulvestrant	
Met BC 10<br>(BB3RC68)	IDC	2	+	ND	-	FEC<br>Capecitabine<br>Paclitaxel	Tamoxifen<br>Fulvestrant	
Met BC 11<br>(BB3RC69)<br>Met BC 11A<br>(BB3RC70)	ILC	2	+	+	-	ECF	Tamoxifen<br>Letrozole<br>Anastrozole<br>Fulvestrant	
Met BC 12<br>(BB3RC71)	IDC	3	+	+	+	FEC<br>Capecitabine<br>Vinorelbine<br>Docetaxel<br>Epirubicin	Tamoxifen<br>Anastrozole<br>Fulvestrant<br>Exemestane	Herceptin<br>Lapatinib
Met BC 13<br>(BB3RC77)	ILC	?	+	+	-	Paclitaxel<br>Capecitabine	Letrozole<br>Exemestane<br>Fulvestrant	
Met BC 14<br>(BB3RC81A)	IDC	2	+	+	-	FEC	Tamoxifen<br>Anastrozole	
Met BC 15<br>(BB3RC82)	IDC	2	+	+	-	TAC<br>5-FU	Tamoxifen<br>Anastrozole<br>Letrozole<br>Goserelin	
Met BC 16<br>(BB3RC83)	IDC	3	+	+	-	FEC<br>Docetaxel<br>Vinorelbine<br>Eribulin	Tamoxifen<br>Anastrozole<br>Fulvestrant	
Met BC 17<br>(BB3RC87)	IDC	3	+	+	-	FEC<br>Paclitaxel<br>Capecitabine<br>Epirubicin<br>Eribulin<br>CMF	Tamoxifen<br>Letrozole<br>Fulvestrant	

IDC: invasive ductal carcinoma; ILC: invasive lobular carcinoma; 5-FU: 5-Fluorouracil; FEC: 5-FU, Epirubicin and Cyclophosphamide; FEC-T: 5-FU, Epirubicin , Cyclophosphamide and Docetaxel; CMF: Cyclophosphamide, Methotrexate and 5-FU; TAC: Docetaxel, Doxorubicin and Cyclophosphamide; ECF: Epirubicin,Cisplatin,5-FU; EOX: Epirubicin, Oxaliplatin, Capecitabine; ND: not determined

NB: ER, PgR and HER-2 were assessed in the primary breast cancer tissue sample.

TABLE S3: Follow-up data from tamoxifen vs. control clinical trial. (Related to Figure 1)

Case Processing Summary

aldh12grx	randomiseringsgrupp	Total N	N of Events	CensoredN	Percent
,00	control	90	51	39	43,3%
	tamoxifen	88	27	61	69,3%
	Overall	178	78	100	**56,2%**
1,00	control	83	37	46	55,4%
	tamoxifen	61	26	35	57,4%
	Overall	144	63	81	**56,2%**
Overall	Overall	322	141	181	56,2%

Overall Comparisons

aldh12grx		Chi-Square	df	Sig.
,00	Log Rank (Mantel-Cox)	10,721	1	,001
1,00	Log Rank (Mantel-Cox)	,261	1	,609

Test of equality of survival distributions for the different levels of randomiseringsgrupp.

Supplemental Experimental Procedures

Patient-derived samples

Primary human breast cancer (BC) tissue was collected from patients at South Manchester, Salford Royal and The Pennine Acute Hospitals NHS Foundation Trusts and metastatic human BC tissue was collected at The Christie NHS Foundation Trust. All patients underwent fully informed consent in accordance with local research ethics committee guidelines (study numbers: 05/Q1402/25 and 05/Q1403/159).

Patient-derived xenografts and *in vivo* experiments

For xenografting the BB3RC31 sample a single cell suspension of 1 million tumor cells in mammosphere media (DMEM/F12 media with L-Glutamine (Gibco) containing B27 supplement (Gibco; 12587) and 20 ng/ml EGF (Sigma)) mixed 1:1 with Matrigel (BD biosciences, 356234) was inoculated subcutaneously into dorsal flanks of NSG mice in a volume of 0.2 ml. BB3RC31 is a pleural effusion from a patient who also presented with liver, lung and abdominal metastasis. For further clinico-pathological details of BB3RC31 please see Table S2. The HBCx34 estrogen-sensitive PDX model and the HBCx22 and HBCx34 tamoxifen-resistant PDX models were kindly provided by Dr Elisabetta Marangoni from Institute Curie, Paris (Cottu et al., 2012; Cottu el al, 2014).

To determine tumor initiation capacity of MCF-7 cells or metastatic (BB3RC31) PDX cells treated *in vitro* or *in vivo*, respectively, NSG mice were injected subcutaneously with cells in mammosphere media mixed 1:1 with Matrigel. 90-day slow release estrogen pellets were implanted sub-cutaneously into mice two days before cell injection (0.72 mg, Innovative Research of America). Serial limiting dilution implantation of MCF-7 cells (10,000; 1,000; 100; 10 cells) and the Extreme Limiting Dilution Analysis (ELDA) software (The Walter and Eliza

Hall Institute of Medical Research) were used to perform calculations (95% CI) of tumor initiation. Positive tumor growth was assessed at day 60 or 90 after cell injection by determining the mice bearing a tumor greater than 100 mm^3 and is represented as mice positive for growth/mice tested (n=4 per condition in all experiments). p values were calculated with Chi-squared test.

Cell lines and culture

MCF7, T47D and ZR-75-1 cell lines were purchased from American Tissue Culture Collection (ATCC) and cultured in DMEM/F-12, GlutaMAX (Gibco) with 10% FBS (Gibco). Cells were treated for 3 days or 6 days with ethanol (control), 10^{-6}M 4-OH-tamoxifen or 10^{-7}M fulvestrant and/or with 10μM of gamma-secretase inhibitor RO4929097 (Cellagen Technology) or DMSO (control).

MCF7 Tamoxifen- and Fulvestrant-resistant cell lines were generated by Dr Julia Gee (University of Cardiff, Wales) and were cultured in phenol red-free DMEM/F12 media with L-Glutamine (Gibco) supplemented with 5% charcoal stripped serum and in the presence of 10^{-7}M 4-OH-tamoxifen or 10^{-7}M fulvestrant, respectively (Knowlden et al., 2003; McClelland et al., 2001). MCF7 resistant cell lines were authenticated by comparative karyotyping to ATCC-derived MCF7.

Mammosphere colony assay

Single cell suspensions of freshly isolated ER+ primary and metastatic patient-derived samples were cultured for 7-9 days (primary cells) in mammosphere colony assays in the presence of ethanol, 10^{-6} M 4-hydroxy-tamoxifen or 10^{-7} M fulvestrant. Primary mammospheres were

dissociated and replated in secondary mammosphere suspension culture for a further 7-9 days to measure self-renewal. Patient-derived xenograft cells were cultured for 7-10 days. For cell lines, cells were pre-treated in adherence and then cultured for 5 days in mammosphere culture (200 cells/cm^2 for cell lines).

Aldefluor assay (Stemcell Technologies)

Dissociated single cells were suspended in Aldefluor assay buffer containing an ALDH substrate, bodipyaminoacetaldehyde (BAAA) at 1.5 mM, and incubated for 45 min at 37ºC. To distinguish between ALDH-positive and -negative cells, a fraction of cells was incubated under identical conditions in the presence of a 2-fold molar excess of the ALDH inhibitor, diethylaminobenzaldehyde (DEAB). Mouse cells were excluded from the FACS analysis with anti-mouse MHC Class I (H-2Kd) antibody conjugated with Pacific Blue (BioLegend, 116616). 7-aminoactinomycin D (7AAD, BD) was added for dead cell exclusion. Data were acquired on a LSR II (BD) flow cytometer and analysed using the BD FACSDiva™ software.

Overexpressing MCF-7 cell lines

Overexpression of Notch IntraCellular Domain 4 (NICD4) or Jagged 1 ligand (JAG1) in MCF-7 cells was carried out by lenti- and retro-viral transduction, respectively.

PCR amplified human NICD4 was inserted in pCDH-EF1-MCS-T2A-puro lentiviral vector (System Biosciences), and puromycin resistance gene was replaced by GFP. Lentiviruses were produced by co-transfection of pPsPax2, pMD2.G vectors and the relevant pCDH lentiviral vector (containing NICD4 or empty vector) in HEK293T cells using polyethylenimine

(Millipore). In order to stimulate lentiviral production 10 mM sodium butyrate was added 8-10 h after transfection. Then, virus-containing supernatant was collected after 48h.

Full length human JAG1 cDNA cloned into the retroviral vector LZRSpBMN-linker-IRES GFP was kindly provided by ML Toribio (CBMSO, Madrid). To obtain retroviral particles, the retroviral vectors (hJAG1-expressing vector or the empty vector) were co-transfected with pMD2.G (envelope system) into PhoenixGP packaging cells following the calcium phosphate method (CalPhos Mammalian Transfection kit, Clontech Laboratories). Retroviruses-containing supernatants were harvested 24, 48 and 72h post-transfection.

Once virus-containing supernatants were collected, they were centrifuged at 400 g for 5min and filtered through 0.45 μm filter to remove cell debris. The viral supernatants were concentrated by ultracentrifugation using an AH-629 rotor (Sorvall Ultra Pro80, Sorvall) at 20000 rpm for 2h at 4°C. The viral pellets were resuspended in PBS and aliquots were stored at -80°C. MCF-7 cells were transduced with either lenti- or retro-viral particles in the presence of 8 μg/ml polybrene (Sigma). Stable transduced MCF-7 cells were selected by FACS based on GFP expression.

All cell lines were cultured at 37°C in 5% CO_2 and experiments were carried out at ~70% confluence.

***NOTCH4* targeting using CRISPR-Cas9n technology**

To generate MCF-7 clones with genomic alterations in *NOTCH4*, a double-nickase approach was used by expressing a D10A mutant version of Cas9 (Cas9 nickase) together with a pair of sgRNAs complementary to opposite strands of the targeted genomic locus. sgRNA oligo sequences (sgRNA-A, 5’-ACCTGCCTGAGCCTGTCTCT-3’; sgRNA-B, 3’-TCTTGGGACACGGTTACCT-5’) targeting exon 2 of *NOTCH4* gene were designed using the

CRISPR design tool (http://crispr.mit.edu/) - **Figure S2D**. sgRNA-coding sequences were cloned into pSpCas9n (BB)-2A-GFP (Addgene, PX461) vector. MCF-7 cells were co-transfected with PX461-sgRNA-A and PX461-sgRNA-B plasmids using X-tremeGENE Transfection reagent (Roche, 06366244001). Two days post-transfection, GFP-positive cells were directly FACS sorted as single cells into 96-well plates. After 14 days in culture, clones were screened for CRISPR-mediated indels. Genomic DNA was extracted using the Wizard Genomic DNA Purification Kit (Promega, A1120) and genotyping PCR was performed using a set of primers flanking the CRISPR target site (forward 5'-GGGTACCATGTGGAGAGTGG-3'; reverse 5'-CACAAGAAGCTGGGTGTCAA-3'). PCR products purified with QIAquick PCR Purification Kit (Qiagen, 28104) were cloned into pGEM-T Easy vector and then Sanger sequenced using T7 sequencing primer. Absence of wild-type alleles was confirmed by using a specific restriction enzyme for target sequence (BanI, Biolabs R0118S). Proteins were extracted and separated on 10% SDS-PAGE gel. Membranes were incubated with anti-NOTCH4 (Santa Cruz, sc-5594) and anti-β-actin (Sigma, A1978) antibodies.

Cancer tissue and cells analysis

Tissue microarrays (TMAs) were constructed from all formalin fixed and paraffin embedded tissue. 3 x 1mm cores were taken per tissue block, and TMAs were cut into 3μm thick sections and analysed by immunohistochemistry. Antibodies utilised were anti-ERα (Thermo, SP1), anti-PgR (Dako, M3569), anti-Ki67 (Dako, M7240), anti-HER2 (Vector Laboratories, VP-C380), anti-HES1 (Abcam, ab108937) and anti-ALDH1 (BD Biosciences, 611195). Antigen retrieval was performed either using Target Retrieval Solution pH9 (Dako S2367, for ER, PgR, Ki67 and ALDH1), Target Retrieval Solution pH6 (Dako S1699, for HES1) or in 10mM Citrate buffer (for

HER2) in a 93^{0}C degree water bath for 25 mins. All antibodies were detected using Dako EnVision Detection System Peroxidase/DAB, Rabbit/Mouse (Dako, K5007) and sections were counterstained with haematoxylin. Staining was quantified using Definiens Tissue Studio software. The percentage of positive epithelial cells was scored on 3 cores per tissue sample.

For ER immunocytochemistry, approximately 50000 cells were FACS sorted and then cytospun for 5 min at 80G on poly-lysine coated slides. Cells were fixed with 4% paraformaldehyde (5 min), permeabilised with acetone (4 min) and methanol (2 min), and then blocked for 10 min with Peroxidase Blocking Reagent (Dako, S2001) and further 10 min with 3% goat serum before incubation with primary antibody. ER was then detected as described above for immunohistochemistry.

Gene Expression Analysis Using Quantitative Real-Time PCR

Total RNA was extracted using the RNeasy Plus Mini Kit (QIAGEN, 74104) and the concentration and purity determined using an ND-1000 spectrophotometer (NanoDrop Technologies). Reverse transcription of 1μg of RNA was performed with Oligo(dT) using the TaqMan Reverse Transcription Reagents from Applied Biosystems (N8080234). Samples were incubated on a thermal cycler (MJ Research) for 10 minutes at 25°C, 30 minutes at 48°C and 5 minutes at 95°C. Quantitative real-time PCR reactions were set up in triplicate in 384-well plates and performed on the 7900 PCR machine (Applied Biosystems) using TaqMan® Universal PCR Master Mix (Applied Biosystems) and probes from Universal Probe Library (Roche). Conditions used for amplification of cDNA fragments were as follows: 95°C for 5 min, 40 cycles of amplification – 95^{0}C for 15 sec, 60^{0}C for 1 min. The expression levels were calculated using the

ΔΔCt method and normalised to the housekeeping genes *36B4* and *GAPDH*. The sequences of the primers and probes used can be found in the table below.

Sequences of primers and probes.

GENE	Forward Primer 5' – 3'	Reverse Primer 5' – 3'	Universal Probe Number
HEY1	CGAGCTGGACGAGACCAT	GAGCCGAACTCAAGTTTCCA	39
HES1	GAAGCACCTCCGGAACCT	GTCACCTCGTTCATGCACTC	60
36B4	TCTACAACCCTGAAGTGCTTGAT	CAATCTGCAGACAGACACTGG	6
GAPDH	AGCCACATCGCTCAGACAC	GCCCAATACGACCAAATCC	60

Protein expression analysis using western-blot

Proteins were extracted with Protein Lysis Buffer (25mM HEPES, 50mM NaCl, 30mM NaPP, 50mM NaF, 1% Triton-X-100, 10% Glycerol, 5mM EDTA, Protease Inhibitor cocktail, 1µM PMSF). Lysates were placed on a rotator for 1 hour at 4°C, then were centrifuged at 10000g at 4°C for 10min, and supernatants were collected. Protein concentrations were assessed using the BCA Protein Assay Kit (Thermo Scientific, 23225). Proteins were separated on a 10% gel (Biorad, 456-1033) by SDS-PAGE at 200V for 1 hour, and then were transferred on polyvinylidene difluoride (PVDF) membranes (BioRad, 170-4157) at 25 V/1,300 mA for 15 min using a Trans Blot Turbo (BioRad, 170-4155). Membranes were blocked in a solution of PBS containing 0,05% Tween-20 and 5% skimmed milk (Marvel) for 1h at room temperature and primary antibodies were incubated overnight at 4°C. Primary antibodies used were anti-NOTCH1 (Rockland, 100401405), anti-NOTCH2 (Cell Signalling, D67C8), anti-NOTCH3 (Santa Cruz, sc-7424), anti-NOTCH4 (Abcam, Ab91621), anti-HES1 (Millipore, AB5702), anti-JAG1 (Santa Cruz, sc-6011), anti-JAG2 (Cell Signalling, 2210), anti-DLL1 (Abcam, Ab76655), anti-DLL3 (Abcam, Ab63707), anti-DLL4 (Abcam, Ab7280) and anti-β-actin (Sigma, A1978).

Horseradish peroxidase-conjugated secondary antibodies (Dako, P0447, P0448, P0449) were incubated for 1h at room temperature. Proteins were visualized with Luminata Classico or Luminata Forte (Millipore, WBLUC0100, WBLUF0100) by exposing the membranes to X-ray films (HyperfilmTM MP, Amersham).

Notch Transcriptional assay

To measure the activation of Notch dependent transcription, cells were transfected with CBF1 firefly luciferase reporter (containing 10 copies of a CBF1 consensus sequence) and CMV-Renilla luciferase reporter. Plasmids were incubated with X-tremegene (Roche, 06366244001) in a ratio of 3:1 (µl of X-tremegene:µg of DNA) in OptiMEM (Life Technologies, 11058-021) for 15 minutes, before addition to the culture media. After 48 hours cells were lysed with 1x Passive lysis buffer (5x, Promega, E 1941), put on the rocker for 15 minutes and luminescence was assayed with the Dual-Glo Luciferase assay system (Promega, E2920) following manufacturer's instructions. Luciferase activity was measured using a luminometer (Promega, Glomax Multi+ Detection System with Instinct Software). Luminescence of the firefly luciferase was normalised to that of the renilla luciferase.

Growth assay (SRB assay)

6000 cells were seeded per well in a 96 well-plate at least in triplicate for each condition used. Plates were incubated in a humidified incubator at 37°C with 5% CO_2 and an SRB assay was performed at different time points to assess cellularity. Briefly, cells were fixed with 25µl/well of 50% (w/v) trichloroacetic (TCA) and incubated at 4°C for a minimum of 1hour. Fixed cells were washed 5 times with water and left to air dry. Cells were then stained with 100µl/well of 0.4%

(w/v) Sulforhodamine B (SRB) dissolved in 1% acetic acid for 30 minutes at room temperature. Residual SRB was washed away with 3 washes of 1% acetic acid and plates were left to air dry. Finally, SRB was solubilized with 100µl/well of 10mM Tris-base (pH 10.5) for 20 minutes at room temperature and absorbance was measured at 490 nm with an automated plate reader (BioTek ELx800).

Notch gene expression signature

Gene expression data was summarised with Ensembl alternative CDF (Dai et al., 2005, Nucleic Acids Res 33:e175) and normalised with RMA, before integration using ComBat (Johnson et al., 2007, Biostatistics; 8: 118–127) to remove dataset-specific bias.

References

Cottu, P., Bieche, I., Assayag, F., El Botty, R., Chateau-Joubert, S., Thuleau, A., Bagarre, T., Albaud, B., Rapinat, A., Gentien, D., et al. (2014). Acquired resistance to endocrine treatments is associated with tumor-specific molecular changes in patient-derived luminal breast cancer xenografts. Clinical cancer research : an official journal of the American Association for Cancer Research 20, 4314-4325.

Cottu, P., Marangoni, E., Assayag, F., de Cremoux, P., Vincent-Salomon, A., Guyader, C., de Plater, L., Elbaz, C., Karboul, N., Fontaine, J.J., et al. (2012). Modeling of response to endocrine therapy in a panel of human luminal breast cancer xenografts. Breast cancer research and treatment 133, 595-606.

Dai, M., Wang, P., Boyd, A.D., Kostov, G., Athey, B., Jones, E.G., Bunney, W.E., Myers, R.M., Speed, T.P., Akil, H., et al. (2005). Evolving gene/transcript definitions significantly alter the interpretation of GeneChip data. Nucleic acids research 33, e175.

Johnson, W.E., Li, C., and Rabinovic, A. (2007). Adjusting batch effects in microarray expression data using empirical Bayes methods. Biostatistics 8, 118-127.

Knowlden, J.M., Hutcheson, I.R., Jones, H.E., Madden, T., Gee, J.M., Harper, M.E., Barrow, D., Wakeling, A.E., and Nicholson, R.I. (2003). Elevated levels of epidermal growth factor receptor/c-erbB2 heterodimers mediate an autocrine growth regulatory pathway in tamoxifen-resistant MCF-7 cells. Endocrinology 144, 1032-1044.

McClelland, R.A., Barrow, D., Madden, T.A., Dutkowski, C.M., Pamment, J., Knowlden, J.M., Gee, J.M., and Nicholson, R.I. (2001). Enhanced epidermal growth factor receptor signaling in MCF7 breast cancer cells after long-term culture in the presence of the pure antiestrogen ICI 182,780 (Faslodex). Endocrinology 142, 2776-2788.
