# Supplementary material for: Anti-estrogen Resistance in Human Breast Tumors Is Driven by JAG1-NOTCH4-Dependent Cancer Stem Cell Activity
Source: Cell Rep. 2015 Sep 17;12(12):1968–77. doi: 10.1016/j.celrep.2015.08.050 (PMC4594158; doi:10.1016/j.celrep.2015.08.050)
Supplement: Document S2. Article plus Supplemental Information [file mmc2.pdf]

Cell Reports

Report

Anti-estrogen Resistance in Human Breast Tumors Is Driven by JAG1-NOTCH4-Dependent Cancer Stem Cell Activity

Graphical Abstract

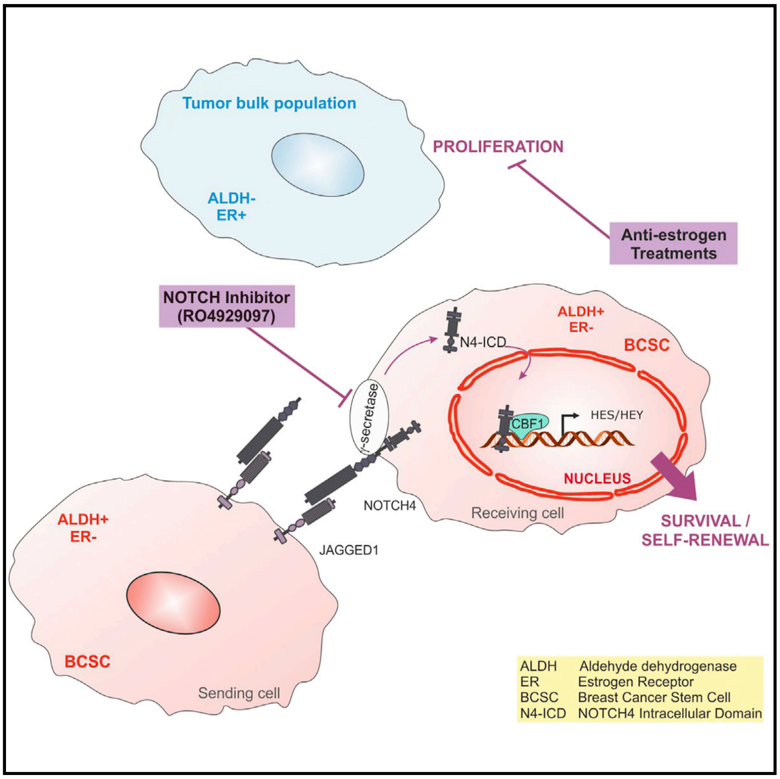

Highlights

- Anti-estrogen therapies selectively enrich for BCSCs and activate Notch signaling
- Notch pathway activation and ALDH1 predict for anti-estrogen treatment failure
- Targeting of Notch4 reduces the population of BCSCs
- Notch inhibitors might prevent relapse or overcome resistance in ER+ tumors

Authors

Bruno M. Simões, Ciara S. O'Brien, Rachel Eyre, ..., Göran Landberg, Sacha J. Howell, Robert B. Clarke

Correspondence

sacha.howell@christie.nhs.uk (S.J.H.), robert.clarke@manchester.ac.uk (R.B.C.)

In Brief

Breast cancers frequently develop resistance to anti-estrogen treatment, which makes it imperative to understanding how therapy resistance develops. Here, Simões et al. show that combining standard anti-estrogen therapies with anti-Notch4 drugs targeting breast cancer stem cells should improve treatment of ER+ breast cancer patients by preventing relapse due to therapy resistance.

CrossMark

Simões et al., 2015, Cell Reports *12*, 1968–1977
September 29, 2015 ©2015 The Authors
http://dx.doi.org/10.1016/j.celrep.2015.08.050

CellPress

OPEN ACCESS
CellPress

Cell Reports
Report

Anti-estrogen Resistance in Human Breast Tumors Is Driven by JAG1-NOTCH4-Dependent Cancer Stem Cell Activity

Bruno M. Simões,[1,9] Ciara S. O'Brien,[1,9] Rachel Eyre,[1] Andreia Silva,[1] Ling Yu,[1] Aida Sarmiento-Castro,[1] Denis G. Alférez,[1] Kath Spence,[1] Angélica Santiago-Gómez,[1] Francesca Chemi,[1,2] Ahmet Acar,[3] Ashu Gandhi,[4] Anthony Howell,[1] Keith Brennan,[3] Lisa Rydén,[5] Stefania Catalano,[2] Sebastiano Andó,[2] Julia Gee,[6] Ahmet Ucar,[1,3] Andrew H. Sims,[7] Elisabetta Marangoni,[8] Gillian Farnie,[1] Göran Landberg,[1] Sacha J. Howell,[1,*] and Robert B. Clarke[1,*]
[1]Breast Cancer Now Research Unit, Institute of Cancer Sciences, University of Manchester, Wilmslow Road, Manchester M20 4BX, UK
[2]Department of Pharmacy, Health and Nutritional Sciences, University of Calabria, 87036 Arcavacata di Rende, Cosenza, Italy
[3]Faculty of Life Sciences, University of Manchester, Oxford Road, Manchester M13 9PT, UK
[4]Manchester Academic Health Science Centre, University Hospital of South Manchester NHS Foundation Trust, Southmoor Road, Manchester M23 9LT, UK
[5]Department of Surgery, Clinical Sciences, Lund University, Skåne University Hospital, 21428 Malmö, Sweden
[6]Cardiff School of Pharmacy and Pharmaceutical Sciences, University of Cardiff, Cardiff, Wales CF10 3NB, UK
[7]Applied Bioinformatics of Cancer Group, Systems Medicine Building, Western General Hospital, University of Edinburgh, Edinburgh EH4 2XU, UK
[8]Laboratoire d'Investigation Préclinique, Institut Curie, 26 rue d'Ulm 75248 Paris Cedex 05, France
[9]Co-first author
*Correspondence: sacha.howell@christie.nhs.uk (S.J.H.), robert.clarke@manchester.ac.uk (R.B.C.)
http://dx.doi.org/10.1016/j.celrep.2015.08.050
This is an open access article under the CC BY license (http://creativecommons.org/licenses/by/4.0/).

SUMMARY

Breast cancers (BCs) typically express estrogen receptors (ERs) but frequently exhibit de novo or acquired resistance to hormonal therapies. Here, we show that short-term treatment with the anti-estrogens tamoxifen or fulvestrant decrease cell proliferation but increase BC stem cell (BCSC) activity through JAG1-NOTCH4 receptor activation both in patient-derived samples and xenograft (PDX) tumors. In support of this mechanism, we demonstrate that high ALDH1 predicts resistance in women treated with tamoxifen and that a NOTCH4/HES/HEY gene signature predicts for a poor response/prognosis in 2 ER+ patient cohorts. Targeting of NOTCH4 reverses the increase in Notch and BCSC activity induced by anti-estrogens. Importantly, in PDX tumors with acquired tamoxifen resistance, NOTCH4 inhibition reduced BCSC activity. Thus, we establish that BCSC and NOTCH4 activities predict both de novo and acquired tamoxifen resistance and that combining endocrine therapy with targeting JAG1-NOTCH4 overcomes resistance in human breast cancers.

INTRODUCTION

Resistance to endocrine therapies such as selective estrogen receptor (ER) modulators (SERMs; e.g., tamoxifen), selective ER downregulators (SERDs; e.g., fulvestrant), and the aromatase inhibitors is seen in 50%–60% of early breast cancer (BC) cases and develops in almost all patients with advanced disease (Davies et al., 2011; Palmieri et al., 2014).

Evidence suggests that tumor-initiating or cancer stem-like cells (CSCs) are responsible for tumor recurrence after chemo- and endocrine therapy (Li et al., 2008; Creighton et al., 2009). Al-Hajj et al. (2003) were the first to show that tumor-initiating cells were capable of recapitulating the original tumor phenotype when transplanted into immunodeficient mice. In vitro functional assays for BC stem cell (BCSC) activity include aldehyde dehydrogenase 1 (ALDH1) enzyme activity and the capacity to form clonogenic mammospheres in suspension culture (Ginestier et al., 2007). It has been demonstrated that the BCSC population is ER negative/low and resistant to the direct effects of endocrine therapy (Simões et al., 2011; Harrison et al., 2013; Piva et al., 2014).

We have shown that aberrant Notch activation transforms normal breast cells, is found in pre-invasive and invasive human BCs, and correlates with early recurrence (Stylianou et al., 2006; Farnie et al., 2007). Moreover, we reported that inhibition of Notch signaling, particularly NOTCH4 receptor, reduced BCSC activity (Harrison et al., 2010).

Here, using patient-derived ER+ BC samples and patient-derived xenografts (PDXs), we report that short-term treatment with endocrine therapies enriches for JAG1-NOTCH4-regulated BCSCs, suggesting that these effects are not through genetic selection. Furthermore, we show that ALDH1 expression and NOTCH4 activation in human primary tumors are predictive of resistance to endocrine treatments. Finally, we demonstrate that NOTCH inhibition in vivo reduces BCSC activity in long-term acquired resistant PDX tumors. Thus, we propose that inhibiting Notch signaling will help overcome endocrine therapy resistance and recurrence in ER+ BC.

OPEN ACCESS
CellPress

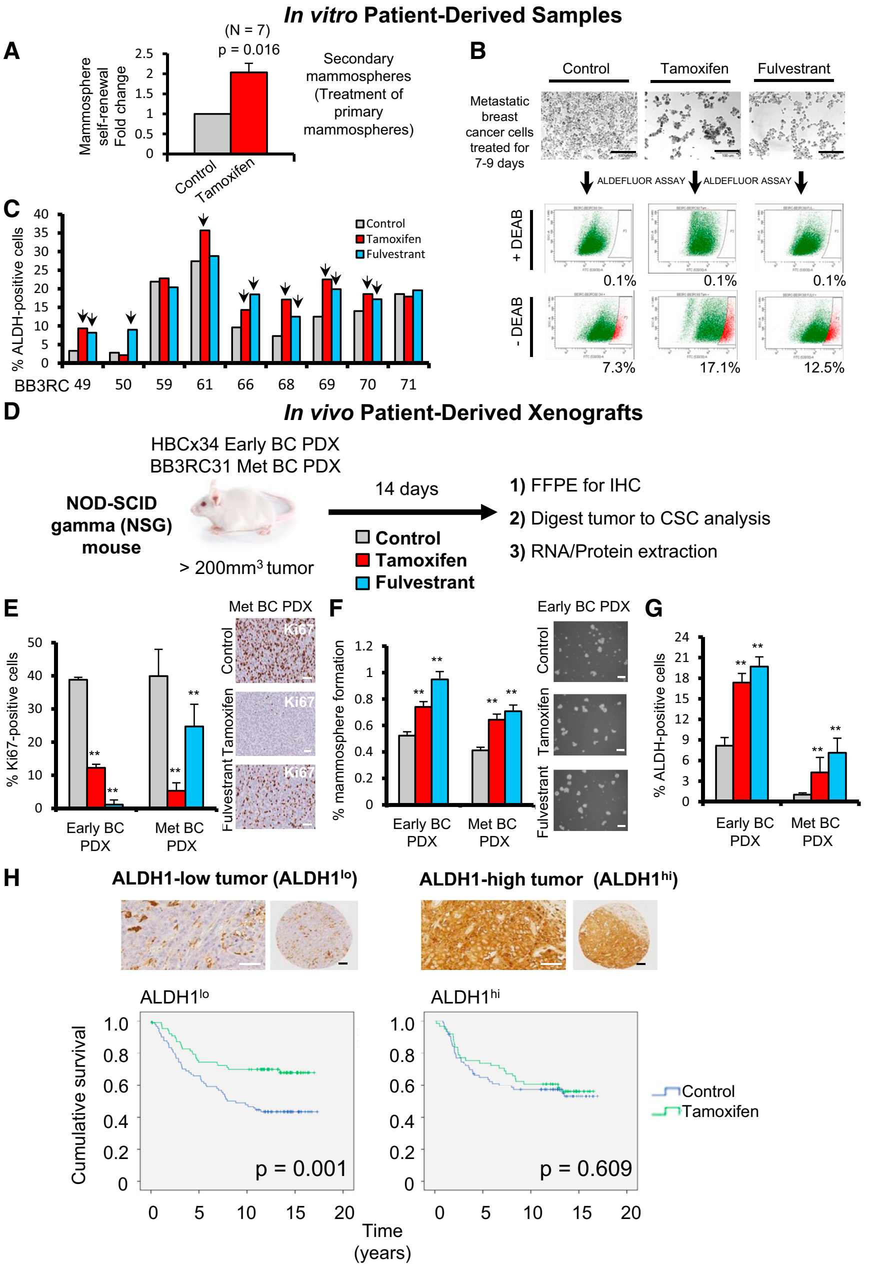

Figure 1. Tamoxifen or Fulvestrant Treatment of ER+ Patient-Derived Samples and PDXs Selectively Enriches for Cells with CSC Properties

High BCSC frequency is associated with worse outcomes for tamoxifen-treated BC patients.

(A) Mammosphere self-renewal of freshly isolated ER+ early and metastatic patient-derived samples. Primary mammospheres cultured in the presence of ethanol (Control) or 10^{-6} M 4-hydroxy-tamoxifen (Tamoxifen) were dissociated and re-plated in secondary mammosphere suspension culture for a further 7–9 days to measure self-renewal of mammosphere-initiating cells treated in the first generation. p value was calculated with Wilcoxon signed-rank test.

(B) Representative micrographs of metastatic BC cells before fluorescence-activated cell sorting (FACS) analysis of ALDH1 enzymatic activity (ALDEFLUOR assay). ALDH-positive cells were discriminated from ALDH-negative cells using the ALDH inhibitor DEAB.

(C) Percentage of ALDH-positive cells in nine ER+ metastatic BC patient-derived samples. Cells were grown in adherence with ethanol (Control), tamoxifen (10^{-6} M), or fulvestrant (10^{-7} M) for 7–9 days. Arrows indicate fold change greater than 20% compared to control.

(D–G) Early (HBCx34) and metastatic (BB3RC31) BC estrogen-dependent PDX tumors treated in vivo for 14 days with tamoxifen (10 mg/kg/day, oral gavage; red bars) or fulvestrant (200 mg/kg/week, subcutaneous injection; blue bars). Gray bars correspond to vehicle control. FFPE, formalin-fixed paraffin-embedded. (E) Representative micrographs and quantification of Ki67 expression determined by immunohistochemistry (IHC). (F) Percentage of MFE. (G) ALDH-positive cells (%) determined using the ALDEFLUOR assay.

(H) ALDH1 expression was assessed by immunohistochemistry in breast tumor epithelial cells, and the percentage of positive cells was scored. Representative micrographs of ALDH-high (ALDH^{hi}) and -low (ALDH^{lo}) epithelial expression are shown. Kaplan-Meier curves represent cumulative survival for the ALDH^{lo} population and ALDH^{hi} population of a cohort of 322 pre-menopausal ER+ BC patients who participated in a randomized trial of 2 years of adjuvant tamoxifen treatment versus no systemic treatment (control). Vertical bars on survival curves indicate censored cases. p values are based on a log-rank (Mantel-Cox) test of equality of survival distributions.

Scale bars, 100 μm. Data are represented as mean ± SEM. $^*p < 0.05$; $^{**}p < 0.01$.

See also Figure S1.

RESULTS

BCSC Activity Is Enriched by Tamoxifen and Fulvestrant

We tested the effect of the anti-estrogen tamoxifen on the mammosphere-forming efficiency (MFE) of patient-derived ER+ tumor cells and found that tamoxifen increases mammosphere self-renewal by about 2-fold (Figures 1A, S1A, and S1B). Next, we investigated ALDH activity, another functional assay for CSCs, in nine patient samples treated with tamoxifen or fulvestrant and showed significant increases in ALDH enzymatic activity in seven patients (Figures 1B and 1C). These data suggest that endocrine therapies, given for a period of a few days, enrich for stem cell activity.

Then, we tested the in vivo impact of endocrine therapies on stem cell activity in ER+ BC using PDXs grown subcutaneously in mice. We used both an early (treatment-naive; early BC) and a metastatic ER+ PDX tumor that both maintain biological characteristics (such as the expression of ER and estrogen

OPEN ACCESS
CellPress

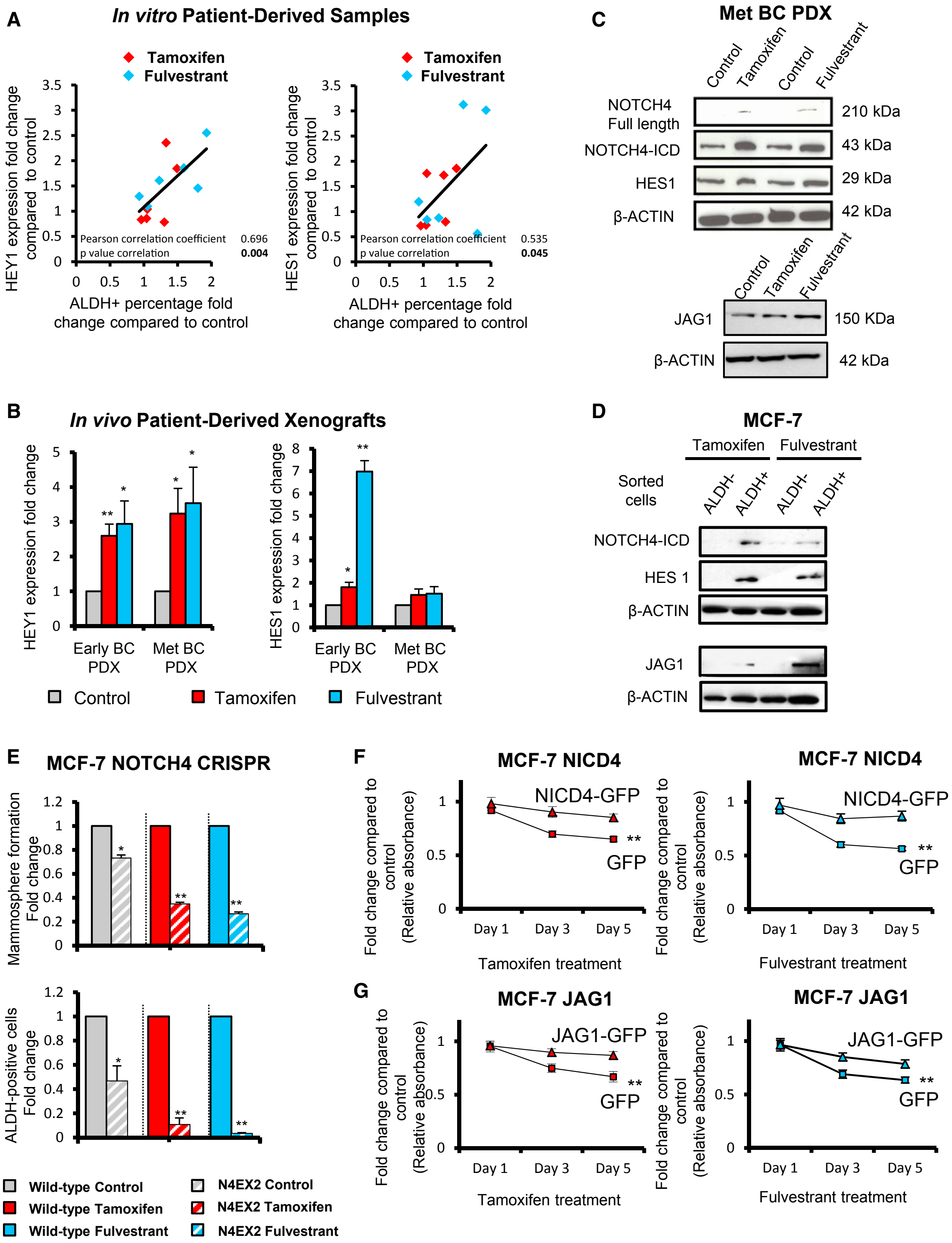

Figure 2. Tamoxifen or Fulvestrant Treatment Upregulates Notch Target Genes in Patient-Derived Samples and PDXs

JAG1-NOTCH4 receptor signaling in ALDH-positive cells drives Notch activity in endocrine-resistant BC.

(A and B) Expression of Notch target genes *HEY1* and *HES1* was assessed by real-time qPCR analysis and compared to control to determine fold change. (A) Metastatic BC patient-derived cells were treated for 7–9 days with ethanol (control), tamoxifen (10^{-6} M), or fulvestrant (10^{-7} M) and a correlation between fold change of expression of HEY1 and HES1 and fold change of percentage of ALDH-positive cells is shown. (B) Early (HBCx34) and metastatic (BB3RC31) BC PDXs: the effect of in vivo treatment for 14 days with tamoxifen (10 mg/kg/day, oral gavage) or fulvestrant (200 mg/kg/week, subcutaneous injection) on *HEY1* and *HES1*.

(legend continued on next page)

dependence) of the patient primary tumor from which they were derived (Figures S1D and S1E). The estrogen dependence of the HBCx34 PDX model (early BC) has been previously reported (Cottu et al., 2012). Using a 14-day in vivo "window" treatment (Figure 1D), we showed that both tamoxifen and fulvestrant treatment decrease proliferation (Figure 1E). However, there is an increase in MFE and ALDH enzymatic activity (Figures 1F and 1G), suggesting a mechanism for endocrine resistance driven by enrichment for a stem cell phenotype.

The mechanism for this enrichment by anti-estrogens may be partly explained by more than 90% of sorted ALDH-positive cells being ER negative (Figure S1C). Thus, we hypothesized that frequency of ALDH-positive cells would predict for response to tamoxifen treatment, and we analyzed ALDH1 in 322 ER+ BC samples taken prior to a randomized trial of tamoxifen versus no systemic treatment. ALDH1 percentage dichotomized at the median value predicted benefit from tamoxifen so that improvement in survival (i.e., a response to treatment) was only seen in women with low epithelial ALDH1 expression (Figure 1H; Table S3). We saw no significant difference in recurrence between control treated patients with high versus low ALDH1 expression ($p = 0.59$). These data, from a prospective randomized trial, establish for the first time that ALDH-positive cell frequency predicts response to tamoxifen treatment, suggesting that stem cell numbers may be responsible for de novo endocrine resistance.

Tamoxifen or Fulvestrant Treatment Upregulates Notch Target Genes

We analyzed the patient-derived BC cells that were treated with tamoxifen and fulvestrant in Figures 1B and 1C and found that increased numbers of ALDH-positive cells were strongly correlated to increased expression of Notch target genes (*HEY1* and *HES1*) (Figure 2A). In addition, the BC PDX tumors treated in vivo with tamoxifen or fulvestrant (Figure 1D) for 2 weeks showed increased *HEY1* and *HES1* expression (Figure 2B), supporting an increased role for the Notch signaling pathway after endocrine therapies.

In ER+ cell lines (MCF-7, T47D, and ZR-75-1) in vitro, treatment with tamoxifen or fulvestrant for 6 days preferentially increased expression of *HEY1* and *HES1* (Figure S2A). Similarly, in tamoxifen-resistant (TAMR) or fulvestrant-resistant (FULVR) MCF-7 models, which have acquired resistance after long-term tamoxifen or fulvestrant treatment, we found upregulation of Notch target genes and increased Notch transcriptional activity (Figure S4A).

JAG1 and NOTCH4 Receptor Signaling Drives Endocrine Resistance

Next, we assessed the expression of Notch receptors and ligands in parental, TAMR, and FULVR cell lines. NOTCH4 and its intracellular domain (ICD) were upregulated while NOTCH1, -2, and -3 were downregulated (Figure S4B) in the resistant versus parental cell lines. We found the Notch ligand JAG1 to be highly expressed in both resistant models (Figure S4B), while expression of the other four ligands was either unchanged (DLL1 and DLL4; Figure S4B) or absent (JAG2 and DLL3; data not shown). JAG1 and NOTCH4-ICD were also upregulated after a 14-day window treatment of PDXs in vivo, and after short-term treatment with tamoxifen or fulvestrant of MCF-7 cells in vitro, suggesting that activation of Notch signaling (demonstrated by increased HES1 expression) is an early event in the acquisition of endocrine resistance (Figures 2C and S2B). Importantly, JAG1, NOTCH4-ICD, and HES1 are expressed at higher levels in ALDH-positive cells, which suggests JAG1-NOTCH4 signaling between ALDH-positive cells (Figure 2D).

To further confirm the role of NOTCH4 activity in endocrine resistance and the stem cell phenotype, we analyzed loss-of- and gain-of-function phenotypes for NOTCH4-ICD in MCF-7 cells. Genomic disruption of exon 2 of *NOTCH4* by using a CRISPR approach led to loss of protein expression (Figures S2C–S2E) and a significant inhibition of MFE and ALDH-positive cells, especially after tamoxifen and fulvestrant treatments (Figure 2E). In contrast, overexpression of NOTCH4-ICD or JAG1 conferred tamoxifen and fulvestrant resistance in parental MCF-7 cells (Figures 2F and 2G).

Overall, these results indicate that JAG1 ligand and cleavage of NOTCH4-ICD may be responsible for Notch signaling activation after endocrine treatment, which is in agreement with recent reports that NOTCH4 expression is increased in TAMR cell lines (Yun et al., 2013; Lombardo et al., 2014).

GSI RO4929097 Abrogates Tamoxifen- and Fulvestrant-Stimulated CSC Activity

In order to inhibit NOTCH4 signaling, we used the gamma-secretase inhibitor (GSI) RO4929097, which we found to be effective in reducing levels of the active NOTCH4 ICD in endocrine-resistant models (Figure S4C). RO4929097 inhibited *HEY1* and *HES1* expression, as well as CBF1-Notch transcriptional activity in TAMR and FULVR cell lines, but not in parental MCF-7 cells (Figure S4D). Therefore, we tested whether RO4929097 would abrogate increases in MFE and ALDH-positive cells induced in vivo by anti-estrogens administered in short-term window

(C) NOTCH4, HES1, and JAG1 protein expression levels determined by western blot in metastatic (Met) (BB3RC31) BC PDX. β-actin was used as a reference for the loading control.

(D) NOTCH4, HES1, and JAG1 protein expression levels were determined by western blot in MCF-7 ALDH-negative and ALDH-positive sorted cells. MCF-7 cells were treated with tamoxifen or fulvestrant for 6 days before ALDH sorting.

(E) Wild-type MCF-7 cells (filled bars) and a CRISPR clone containing a disruption of *NOTCH4* exon 2 (N4EX2 cells, hatched bars) treated in adherence with ethanol (Control, gray bars), 10^{-6} M tamoxifen (red bars), and 10^{-7} M fulvestrant (blue bars) for 6 days. N4EX2 cells' fold change of MFE and ALDH-positive cells after treatments was compared to that of the wild-type cells.

(F and G) NICD4 and JAG1 rescue tamoxifen- or fulvestrant-inhibited growth: cell number (using sulforhodamine B [SRB] assay, y axis) of MCF-7 overexpressing (F) NICD4-GFP, (G) JAG1-GFP, or GFP control incubated with tamoxifen or fulvestrant for 1, 3, and 5 days (x axis) compared to the respective cell line treated with control ethanol. p values are for the 5-day treatment.

Data are represented as mean ± SEM. $^{*}p < 0.05$; $^{**}p < 0.01$.

See also Figures S2 and S4.

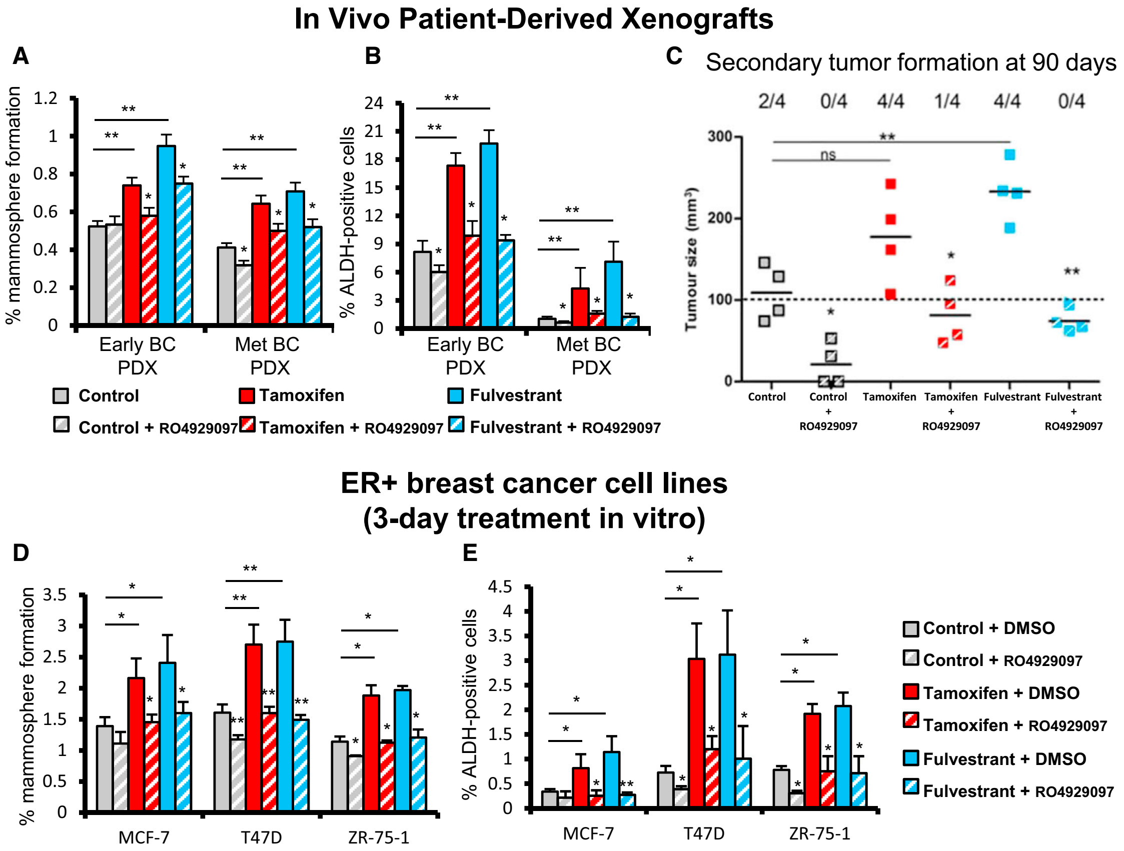

F

MCF-7
(6-day treatment in vitro)

MCF-7 cells 6-day pre-treatment groups		Control + DMSO	Control + RO4929097	Tamoxifen + DMSO	Tamoxifen + RO4929097	Fulvestrant + DMSO	Fulvestrant + RO4929097
Positive tumor growth from different cell numbers	10 000 cells	4/4	3/4	4/4	4/4	4/4	4/4
	1 000 cells	3/4	1/4	4/4	3/4	4/4	2/4
	100 cells	0/4	0/4	2/4	1/4	2/4	1/4
	10 cells	0/4	0/4	2/4	0/4	2/4	0/4
Tumor Initiating Cell Frequency (95% CI)		**1:888** (1:282-1:2801)	**1:6276** (1:2115-1:18619)	**1:77** (1:25-1:239)	**1:626** (1:211-1:1861)	**1:77** (1:25-1:239)	**1:1078** (1:325-1:3572)
P from Control + DMSO group (χ^2)		N/A	N/A	0.0025	N/A	0.0025	N/A
P from respective DMSO group (χ^2)		N/A	0.0205	N/A	0.0070	N/A	0.0009

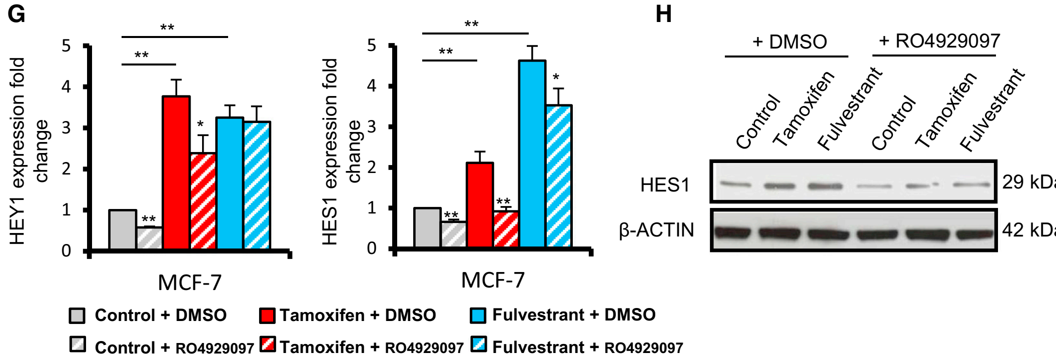

(legend on next page)

treatments, using the same estrogen-dependent ER+ PDX tumors as in Figures 1D–1G. Tamoxifen and fulvestrant treatments reduced tumor growth and proliferation (Figures S3A and S3B) while increasing both MFE and ALDH activity (Figures 3A and 3B). RO4929097 had no impact on growth or proliferation (% Ki67; Figure S3B) but significantly inhibited endocrine-stimulated MFE and ALDH activity (Figures 3A and 3B). The gold standard for functionally determining tumor-initiating cells is xenograft formation in secondary mouse hosts, which we performed using dissociated cells from PDX tumors treated in vivo with anti-estrogens and/or RO4929097. Cells isolated from tumors treated in vivo with RO4929097 had significantly reduced tumor-initiating capacity 90 days post-implantation (Figure 3C). Furthermore, the stimulation of tumorigenicity following in vivo tamoxifen and fulvestrant treatment was completely reversed by RO4929097 (Figure 3C). Overall, these data suggest that BCSCs, measured by tumor-initiating activity and enriched by short-term anti-estrogen treatments, are dependent on NOTCH4 signaling that can be blocked by combination treatment with a NOTCH4 inhibitor.

To further substantiate this finding, we analyzed MFE and ALDH activity of MCF-7, T47D, and ZR-75-1 cells treated for 3 days with tamoxifen or fulvestrant in combination with RO4929097. In all cases, RO4929097 reduced MFE and ALDH-positive cells (Figures 3D and 3E). To confirm that RO4929097 reduced the tumor-initiating capacity, we conducted in vivo limiting dilution transplantation of MCF-7 cells. Extreme limiting dilution analysis (ELDA) revealed an 11-fold enrichment in tumor-initiating cell frequency following tamoxifen or fulvestrant pre-treatment, which was reversed by co-treatment with RO4929097 (Figure 3F). Inhibition of NOTCH4 cleavage/activation by RO4929097 was evidenced by decreased HEY1 and HES1 mRNA and protein levels (Figures 3G and 3H). Thus, we established, using PDX models and cell lines in tumor-initiating cell assays, that NOTCH4 inhibition reduces BCSC activity induced by anti-estrogen treatment.

NOTCH4 Inhibition Targets CSCs in TAMR PDX Models

The next question we asked was whether inhibiting NOTCH4 signaling to target BCSCs will overcome long-term acquired anti-estrogen resistance in ER+ BC patients. We investigated RO4929097 treatment in two established PDXs (HBCx22 and HBCx34) that have long-term acquired resistance to tamoxifen in vivo. Analysis of HES1 expression by immunohistochemistry revealed that these two TAMR PDXs displayed increased Notch signaling activation compared to the parental control (Figure 4A). Notably, the TAMR HBCx34 PDX model has a higher percentage of MFE and ALDH activity than the endocrine-sensitive HBCx34 PDX model (compare Figures 1F and 1G with Figures 4B and 4C). These data suggest that acquired tamoxifen resistance in PDX models involves enrichment for BCSC activity through Notch signaling. Treatment with RO4929097 for 14 days demonstrates that MFE and ALDH activity can be significantly reduced in TAMR PDX tumors in vivo (Figures 4B–4D).

NOTCH4/HES/HEY Gene Signature Predicts for Resistance to Tamoxifen Treatment and Prognosis in ER+ Tumors

Based on the aforementioned observations, we hypothesized that NOTCH4 activity, comprising a NOTCH4/HES/HEY gene signature, would predict for response to tamoxifen treatment. In gene expression data from 669 pre-treatment tumors from four published Affymetrix microarray datasets of ER+ patients who subsequently received adjuvant tamoxifen therapy, we found *NOTCH4*, *HES1*, *HEY1*, and *HEY2* to be co-expressed in some tumors, as demonstrated in the heatmap ordered from left to right by the sum of the four genes (Figure 5A). Importantly, elevated expression of these Notch genes before treatment was significantly associated with distant metastasis (Figure 5A) and with reduced overall survival in an independent cohort of 343 untreated ER+ patients (Figure 5B). Thus, *NOTCH4* gene expression and activity in tumors before treatment with endocrine therapy predicts sensitivity to treatment, indicating that this signaling pathway predicts de novo as well as acquired endocrine resistance. These data strengthen the case for therapies against NOTCH4 to target the endocrine-resistant ALDH-positive cells responsible for relapse of ER+ tumors following hormonal therapy (Figure 5C).

DISCUSSION

Here, we report that BCSC activity and frequency are increased in response to the common endocrine therapies tamoxifen and fulvestrant in ER+ patient samples and in early and metastatic PDXs. Our findings suggest that endocrine therapies do not target BCSCs, and this may explain how residual drug-resistant cells are responsible for the relapse of ER+ tumors following hormonal therapy. Although we observe increased BCSC frequency after endocrine treatments, we do not know whether absolute BCSC numbers remain the same and are selected for or whether

Figure 3. NOTCH4 Inhibition using RO4929097 Abrogates Tamoxifen and Fulvestrant Enrichment of CSC Activities

(A–C) Early (HBCx34) and metastatic (Met) (BB3RC31) PDX tumors treated in vivo for 14 days with tamoxifen (10 mg/kg/day, oral gavage) or fulvestrant (200 mg/kg/week, subcutaneous injection) in the presence or absence of the NOTCH4 inhibitor RO4929097 (3 mg/kg/day, oral gavage). (A) MFE (%). (B) Percentage of ALDH-positive cells. (C) Secondary tumor formation. 100,000 cells of metastatic (BB3RC31) PDX were re-implanted subcutaneously in NSG mice with 90-day slow-release estrogen pellets. Tumor growth (>100 mm^3) was determined at day 90 after cell injection.

(D and E) MCF-7, T47D, and ZR-75-1 cells were pre-treated in adherence with 10^{-6} M tamoxifen (red bars) and 10^{-7} M fulvestrant (blue bars) with RO4929097 (10 μM; hatched bars) or DMSO (filled bars) for 72 hr. (D) MFE and (E) percentage of ALDH-positive cells were assessed after pre-treatments.

(F–H) MCF-7 cells were pre-treated in adherence for 6 days in the presence of RO4929097 (10 μM; hatched bars) or DMSO (filled bars). (F) In vivo experiments were carried out in NSG mice with 90-day slow-release estrogen pellets. Tumor growth (>100 mm^3) was assessed at day 60 and is represented as mice positive for growth/mice tested for each cell number tested. ELDA of tumor-initiating cell frequency is shown. (G) Expression of *HEY1* and *HES1* by real-time PCR was compared to the control. (H) HES1 protein expression levels determined by western blot.

Data are represented as mean ± SEM. p values refer to hatched bars compared to filled control bars. *$p < 0.05$; **$p < 0.01$.

See also Figures S3 and S4.

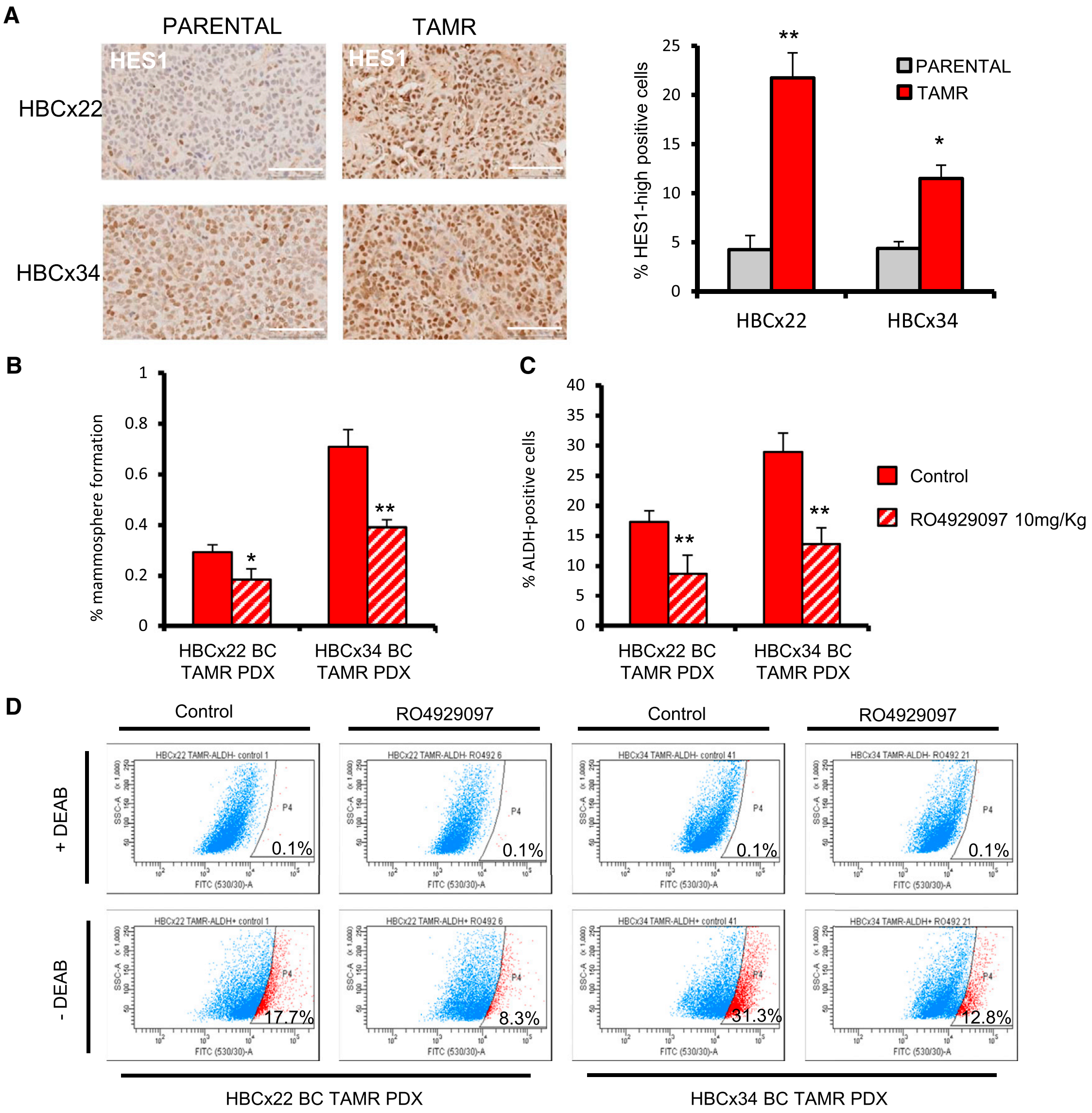

Figure 4. HBCx22 and HBCx34 TAMR PDXs Express High Levels of HES1

NOTCH4 inhibitor RO4929097 targets CSCs in tamoxifen-resistant (TAMR) PDXs.

(A) Representative micrographs and quantification of HES1 expression determined by immunohistochemistry. Scale bars, 100 μm.

(B–D) HBCx22 and HBCx34 TAMR PDXs treated in vivo for 14 days in the presence or absence of the GSI RO4929097 (10 mg/kg/day, oral gavage). (B) MFE (%). (C) Percentage of ALDH-positive cells. (D) Representative FACS plots of ALDEFLUOR assay. ALDH-positive cells were discriminated from ALDH-negative cells using the ALDH inhibitor DEAB.

Data are represented as mean ± SEM. *$p < 0.05$; **$p < 0.01$.

See also Figure S4.

they can be induced by anti-estrogen treatment. Tamoxifen and fulvestrant are clearly successful in reducing BC recurrence in some patients. In other patients with poorer outcome after endocrine therapies, we demonstrate that tumors have high pre-treatment levels of ALDH1 expression and NOTCH4 activation. Moreover, we found that treating ER+ BC cells with endocrine therapies specifically increases JAG1-NOTCH4 signaling and that combining endocrine therapies with a Notch pathway inhibitor can prevent BCSC enrichment induced by endocrine therapies. Thus, our findings in patient-derived BCSCs establish that JAG1 ligand signaling through the NOTCH4 receptor in ALDH-positive cell populations is a determining factor in the acquisition of endocrine resistance.

The best described strategy for inhibition of Notch signaling is the use of small-molecule GSIs, which prevent the release of Notch ICD (NICD). In our study, the GSI RO4929097 specifically targets NOTCH4 cleavage in anti-estrogen-treated cells and, thus, decreases BCSC activity in vitro (MFE and ALDH activity)

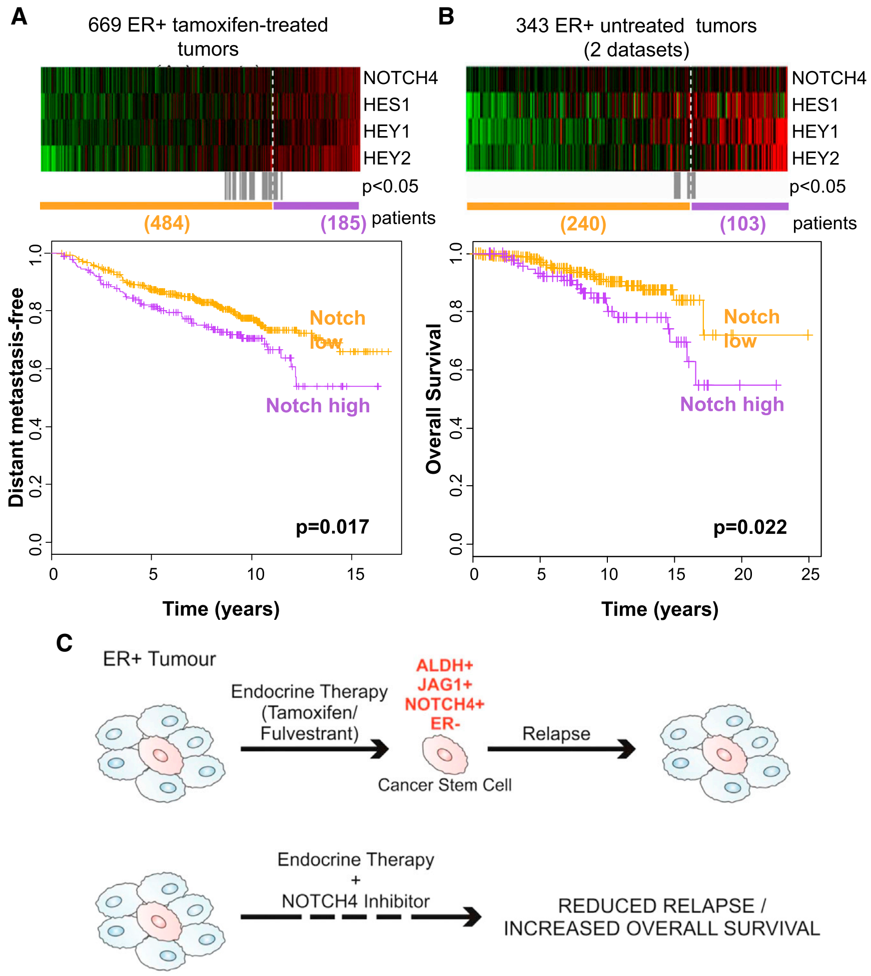

Figure 5. NOTCH4 Receptor Activity Predicts for Resistance to Tamoxifen Treatment and Prognosis in ER+ Tumors

(A and B) *NOTCH4*, *HES1*, *HEY1*, and *HEY2* genes in ER+ primary tumors from (A) tamoxifen-treated or (B) untreated patients are co-expressed in the heatmap ranked from left to right using the four-gene signature. Colors are $\log_2$ mean-centered values; red indicates high, and green indicates low. All significant cut-points ($p < 0.05$) are shown in gray. Kaplan-Meier analysis using the optimum cut-point (dashed white line) demonstrates that elevated expression of the Notch genes is significantly associated with an increased rate of (A) distant metastasis and (B) decreased overall survival. Vertical bars on survival curves indicate censored cases. p values are based on a log-rank (Mantel-Cox) test.

(C) Diagram suggesting that endocrine therapies do not target BCSCs and emphasizing the need of targeting residual drug-resistant cells to eliminate all cancer cells and prevent long-term recurrences of ER+ BC.

and tumor initiation in vivo. Our investigations in ER+ PDX tumors provide the rationale for the use of NOTCH4 inhibitors together with endocrine therapies in the adjuvant or advanced settings (Figure 5C). Significantly, we demonstrated the utility of RO4929097 to target BCSCs in pre-clinical models of TAMR patient tumors.

In conclusion, our data establish that tamoxifen and fulvestrant select for stem cell activity in short- and long-term-treated BC cells, as well as in early endocrine therapy naive and metastatic-endocrine-treated patient-derived samples and PDXs. Importantly, we report that low numbers of stem cells and low Notch signaling activation in patient tumors predict response to tamoxifen therapy and better survival. Overall, these results suggest that ER+ BC recurrence after endocrine therapies, which target the majority of cells (ER+ cells), will be reduced by targeting the JAG1+/NOTCH4+/ALDH1+/ER− BCSC population.

EXPERIMENTAL PROCEDURES

Patient-Derived Samples

Early BC samples were collected in RPMI (GIBCO), dissected into 1- to 2-mm^3 cubes and digested with the Human Tumor Dissociation Kit (Miltenyi Biotec) for 2 hr at 37°C. Digested tissue was filtered sequentially through 100- and 40-μm cell strainers, then centrifuged at 300 × g for 5 min and washed in PBS.

Metastatic samples (ascites or pleural effusions) were centrifuged at 1,000 × g for 10 min at 4°C. The cell pellets were diluted in PBS. Erythrocytes and leucocytes were removed using Lymphoprep (Axis-Shield) and CD45-negative magnetic sorting (Miltenyi Biotec), respectively. Cells were cultured in adherence for 7–9 days in DMEM/F-12 medium, GlutaMAX (GIBCO) with 10% fetal bovine serum (FBS; GIBCO), 10 μg/ml insulin (Sigma-Aldrich), 10 μg/ml hydrocortisone (Sigma-Aldrich), and 5 ng/ml epidermal growth factor (EGF; Sigma-Aldrich), in 10^{-6} M 4-OH tamoxifen (Sigma-Aldrich, H7904), 10^{-7} M fulvestrant (ICI 182,780, Tocris, 1047), or ethanol (control).

Clinico-pathological details of the samples are summarized in Tables S1 (primary BC) and S2 (metastatic BC).

Please refer to the Supplemental Experimental Procedures for further details.

PDXs and In Vivo Experiments

Mouse studies commenced in 8- to 12-week-old female mice and were conducted in accordance with the UK Home Office Animals (Scientific Procedures) Act 1986, using NSG (NOD.Cg-$Prkdc^{scid}$ $Il2rg^{tm1Wjl}$/SzJ) mice. All in vivo work was performed with a minimum of n = 4 mice per condition.

Serial passaging of the PDX was carried out by implanting small fragments of the tumor subcutaneously into dorsal flanks of NSG mice. Early (HBCx34) and metastatic (BB3RC31) BC estrogen-dependent PDXs were administered with 8 μg/ml of 17-beta estradiol in drinking water at all times and were treated with drugs when tumors reached 200–300 mm^3. Experiments were performed using PDX tumors between passages 5 and 8. Animal weight and tumor size was measured bi-dimensionally using callipers twice a week.

Tamoxifen citrate (Sigma, T9262, 10 mg/kg/day) and RO4929097 (Cellagen Technology, 3 mg/kg/day) were administered by oral gavage (0.1 ml per dose) on a basis of 5 days out of 7 (weekends excluded) for 14 days. Tamoxifen citrate and RO4929097 were prepared in 1% carboxymethylcellulose (Sigma, C9481) dissolved in distilled water. Fulvestrant (kindly provided by AstraZeneca, 200 mg/kg/week) was administered by subcutaneous injection

(0.1 ml per dose) on a weekly basis for 14 days. The HBCx22 and HBCx34 TAMR PDXs were treated for 14 days in the presence or absence of the GSI RO4929097 (10 mg/kg/day, oral gavage). Xenografts were collected in ice-cold DMEM for live-cell assays, histological analysis, and RNA and protein extraction. PDX single-cell suspension was obtained using a collagenase-hyaluronidase mixture for digestion (Stem Cell Technologies).

Please refer to the Supplemental Experimental Procedures for further details.

Mammosphere Colony Assay

MFE was calculated by dividing the number of mammospheres formed ($\geq$50 μm) by the original number of single cells seeded (500 cells per square centimeter for primary cells) and is expressed as fold change normalized to control or as the mean percentage of MFE (Shaw et al., 2012).

Please refer to the Supplemental Experimental Procedures for further details.

Tamoxifen Trial Study

Premenopausal BC patients with invasive stage II disease were enrolled in SBII:2a, a Swedish clinical trial in which patients were randomly assigned to receive 2 years of adjuvant tamoxifen or no treatment (control) and followed up for recurrence-free and overall survival (Rydén et al., 2005). Our data represent cumulative survival for a cohort of 322 premenopausal ER+ BC patients stratified by ALDH-low (below median) and ALDH-high (above median) expression over time.

Notch Gene Expression Signature

The gene expression data on 669 ER+ tamoxifen-treated tumors (GSE6532, GSE9195, GSE17705, and GSE12093) and 343 ER+ untreated tumors (GSE2034 and GSE7390) are from published Affymetrix microarray datasets.

Please refer to the Supplemental Experimental Procedures for additional details.

Statistical Analysis

If not stated otherwise, a two-tailed Student's t test was performed for statistical analysis. A value of $p < 0.05$ was considered to be statistically significant. Error bars represent the SEM of at least three independent experiments. Data are shown as mean ± SEM.

SUPPLEMENTAL INFORMATION

Supplemental Information for this article includes Supplemental Experimental Procedures, four figures, and three tables and can be found with this article online at http://dx.doi.org/10.1016/j.celrep.2015.08.050.

AUTHOR CONTRIBUTIONS

B.M.S. and C.S.O.: conception and design, collection and/or assembly of data, data analysis and interpretation, and manuscript writing. R.E., A.S., L.Y., A.S.-C., D.G.A., K.S., A.S.-G., F.C., A.A., S.C., S.A., A.U., and G.F.: collection and/or assembly of data, data analysis and interpretation, and manuscript critique. A.G.: study recruitment, collection of BC tissue, and manuscript critique. A.H. and K.B.: conception, design, and manuscript critique. J.G.: generation of resistant cell lines and manuscript critique. L.R. and G.L.: Tamoxifen trial study and BC clinical pathology expertise and manuscript critique. A.H.S.: gene expression analysis and manuscript critique. E.M.: generation of PDX and manuscript critique. S.J.H. and R.B.C.: conception and design, data analysis and interpretation, manuscript writing, and final approval of manuscript.

ACKNOWLEDGMENTS

We are a Breast Cancer Now-funded research group (Institute of Cancer Sciences, University of Manchester). We are grateful for funding from the Cancer Research UK (C.S.O.), Medical Research Council (A.S.-C.), Fundación Alfonso Martín Escudero (A.S.-G.), and MultiFun EU FP7 grant #262943 (B.M.S. and K.S.). We thank Allan Jordan and Rebecca Newton (Drug Discovery Unit, Cancer Research UK Manchester Institute) for advice regarding small-molecule Notch inhibitors and Anne Lykkesfeldt for advice on the use of TAMR MCF-7 cells (Danish Cancer Society Research Center). We acknowledge the South-East Swedish Breast Cancer Group for providing access to clinical data and tumor tissue.

Received: March 17, 2015
Revised: July 15, 2015
Accepted: August 17, 2015
Published: September 17, 2015

REFERENCES

Al-Hajj, M., Wicha, M.S., Benito-Hernandez, A., Morrison, S.J., and Clarke, M.F. (2003). Prospective identification of tumorigenic breast cancer cells. Proc. Natl. Acad. Sci. USA *100*, 3983–3988.

Cottu, P., Marangoni, E., Assayag, F., de Cremoux, P., Vincent-Salomon, A., Guyader, Ch., de Plater, L., Elbaz, C., Karboul, N., Fontaine, J.J., et al. (2012). Modeling of response to endocrine therapy in a panel of human luminal breast cancer xenografts. Breast Cancer Res. Treat. *133*, 595–606.

Creighton, C.J., Li, X., Landis, M., Dixon, J.M., Neumeister, V.M., Sjolund, A., Rimm, D.L., Wong, H., Rodriguez, A., Herschkowitz, J.I., et al. (2009). Residual breast cancers after conventional therapy display mesenchymal as well as tumor-initiating features. Proc. Natl. Acad. Sci. USA *106*, 13820–13825.

Davies, C., Godwin, J., Gray, R., Clarke, M., Cutter, D., Darby, S., McGale, P., Pan, H.C., Taylor, C., Wang, Y.C., et al.; Early Breast Cancer Trialists' Collaborative Group (EBCTCG) (2011). Relevance of breast cancer hormone receptors and other factors to the efficacy of adjuvant tamoxifen: patient-level meta-analysis of randomised trials. Lancet *378*, 771–784.

Farnie, G., Clarke, R.B., Spence, K., Pinnock, N., Brennan, K., Anderson, N.G., and Bundred, N.J. (2007). Novel cell culture technique for primary ductal carcinoma in situ: role of Notch and epidermal growth factor receptor signaling pathways. J. Natl. Cancer Inst. *99*, 616–627.

Ginestier, C., Hur, M.H., Charafe-Jauffret, E., Monville, F., Dutcher, J., Brown, M., Jacquemier, J., Viens, P., Kleer, C.G., Liu, S., et al. (2007). ALDH1 is a marker of normal and malignant human mammary stem cells and a predictor of poor clinical outcome. Cell Stem Cell *1*, 555–567.

Harrison, H., Farnie, G., Howell, S.J., Rock, R.E., Stylianou, S., Brennan, K.R., Bundred, N.J., and Clarke, R.B. (2010). Regulation of breast cancer stem cell activity by signaling through the Notch4 receptor. Cancer Res. *70*, 709–718.

Harrison, H., Simoes, B.M., Rogerson, L., Howell, S.J., Landberg, G., and Clarke, R.B. (2013). Oestrogen increases the activity of oestrogen receptor negative breast cancer stem cells through paracrine EGFR and Notch signalling. Breast Cancer Res. *15*, R21.

Li, X., Lewis, M.T., Huang, J., Gutierrez, C., Osborne, C.K., Wu, M.F., Hilsenbeck, S.G., Pavlick, A., Zhang, X., Chamness, G.C., et al. (2008). Intrinsic resistance of tumorigenic breast cancer cells to chemotherapy. J. Natl. Cancer Inst. *100*, 672–679.

Lombardo, Y., Faronato, M., Filipovic, A., Vircillo, V., Magnani, L., and Coombes, R.C. (2014). Nicastrin and Notch4 drive endocrine therapy resistance and epithelial to mesenchymal transition in MCF7 breast cancer cells. Breast Cancer Res. *16*, R62.

Palmieri, C., Patten, D.K., Januszewski, A., Zucchini, G., and Howell, S.J. (2014). Breast cancer: current and future endocrine therapies. Mol. Cell. Endocrinol. *382*, 695–723.

Piva, M., Domenici, G., Iriondo, O., Rábano, M., Simões, B.M., Comaills, V., Barredo, I., López-Ruiz, J.A., Zabalza, I., Kypta, R., and Vivanco, Md. (2014). Sox2 promotes tamoxifen resistance in breast cancer cells. EMBO Mol. Med. *6*, 66–79.

Rydén, L., Jönsson, P.E., Chebil, G., Dufmats, M., Fernö, M., Jirström, K., Källström, A.C., Landberg, G., Stål, O., Thorstenson, S., et al. (2005). Two years of adjuvant tamoxifen in premenopausal patients with breast cancer: a randomised, controlled trial with long-term follow-up. Eur. J. Cancer *41*, 256–264.

Shaw, F.L., Harrison, H., Spence, K., Ablett, M.P., Simões, B.M., Farnie, G., and Clarke, R.B. (2012). A detailed mammosphere assay protocol for the quantification of breast stem cell activity. J. Mammary Gland Biol. Neoplasia *17*, 111–117.

Simões, B.M., Piva, M., Iriondo, O., Comaills, V., López-Ruiz, J.A., Zabalza, I., Mieza, J.A., Acinas, O., and Vivanco, M.D. (2011). Effects of estrogen on the proportion of stem cells in the breast. Breast Cancer Res. Treat. *129*, 23–35.

Stylianou, S., Clarke, R.B., and Brennan, K. (2006). Aberrant activation of notch signaling in human breast cancer. Cancer Res. *66*, 1517–1525.

Yun, J., Pannuti, A., Espinoza, I., Zhu, H., Hicks, C., Zhu, X., Caskey, M., Rizzo, P., D'Souza, G., Backus, K., et al. (2013). Crosstalk between PKCα and Notch-4 in endocrine-resistant breast cancer cells. Oncogenesis *2*, e60.

Cell Reports

Supplemental Information

Anti-estrogen Resistance in Human Breast Tumors Is Driven by JAG1-NOTCH4-Dependent Cancer Stem Cell Activity

Bruno M. Simões, Ciara S. O'Brien, Rachel Eyre, Andreia Silva, Ling Yu, Aida Sarmiento-Castro, Denis G. Alférez, Kath Spence, Angélica Santiago-Gómez, Francesca Chemi, Ahmet Acar, Ashu Gandhi, Anthony Howell, Keith Brennan, Lisa Rydén, Stefania Catalano, Sebastiano Andó, Julia Gee, Ahmet Ucar, Andrew H. Sims, Elisabetta Marangoni, Gillian Farnie, Göran Landberg, Sacha J. Howell, and Robert B. Clarke

Supplementary figure 1

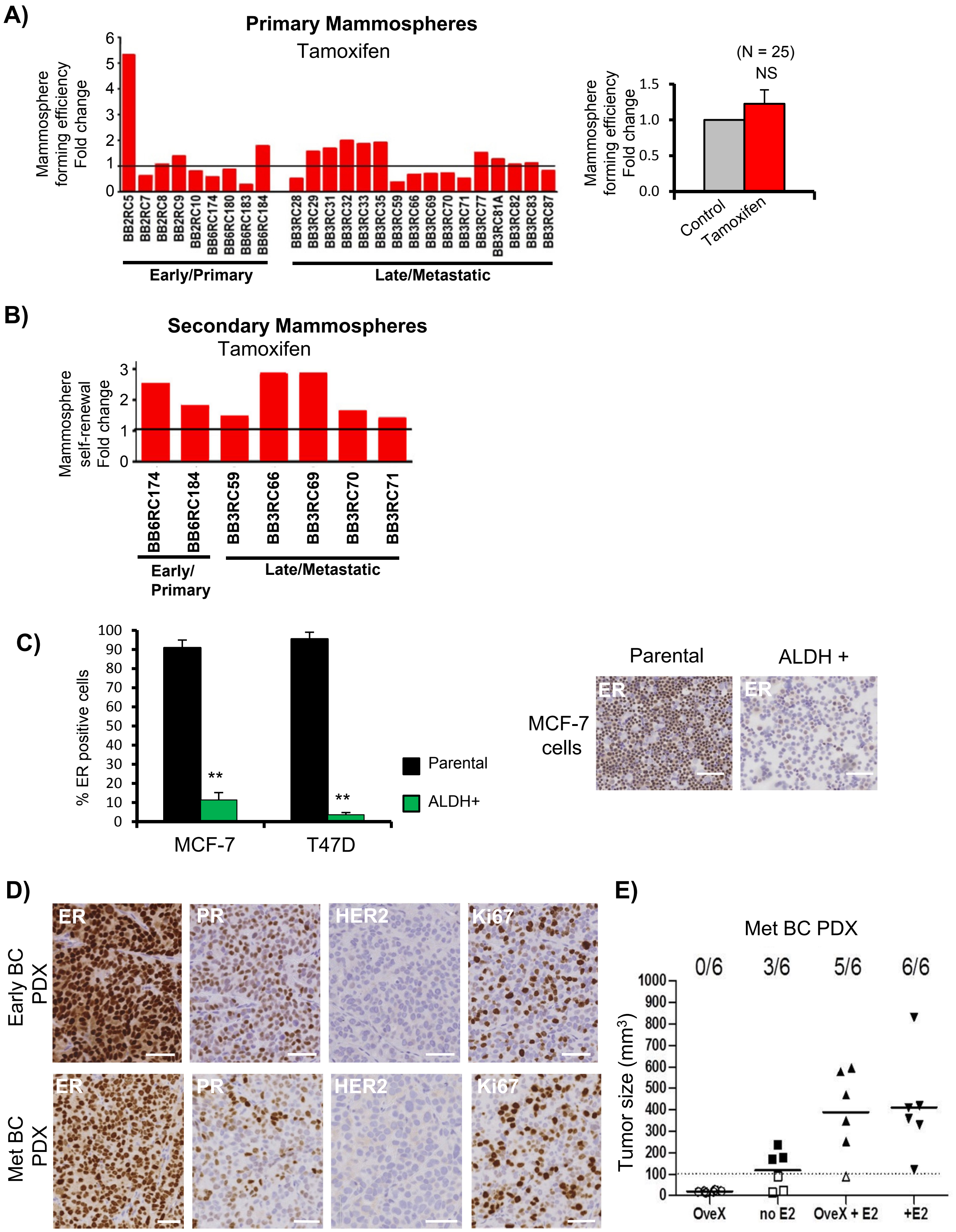

Supplementary figure 2

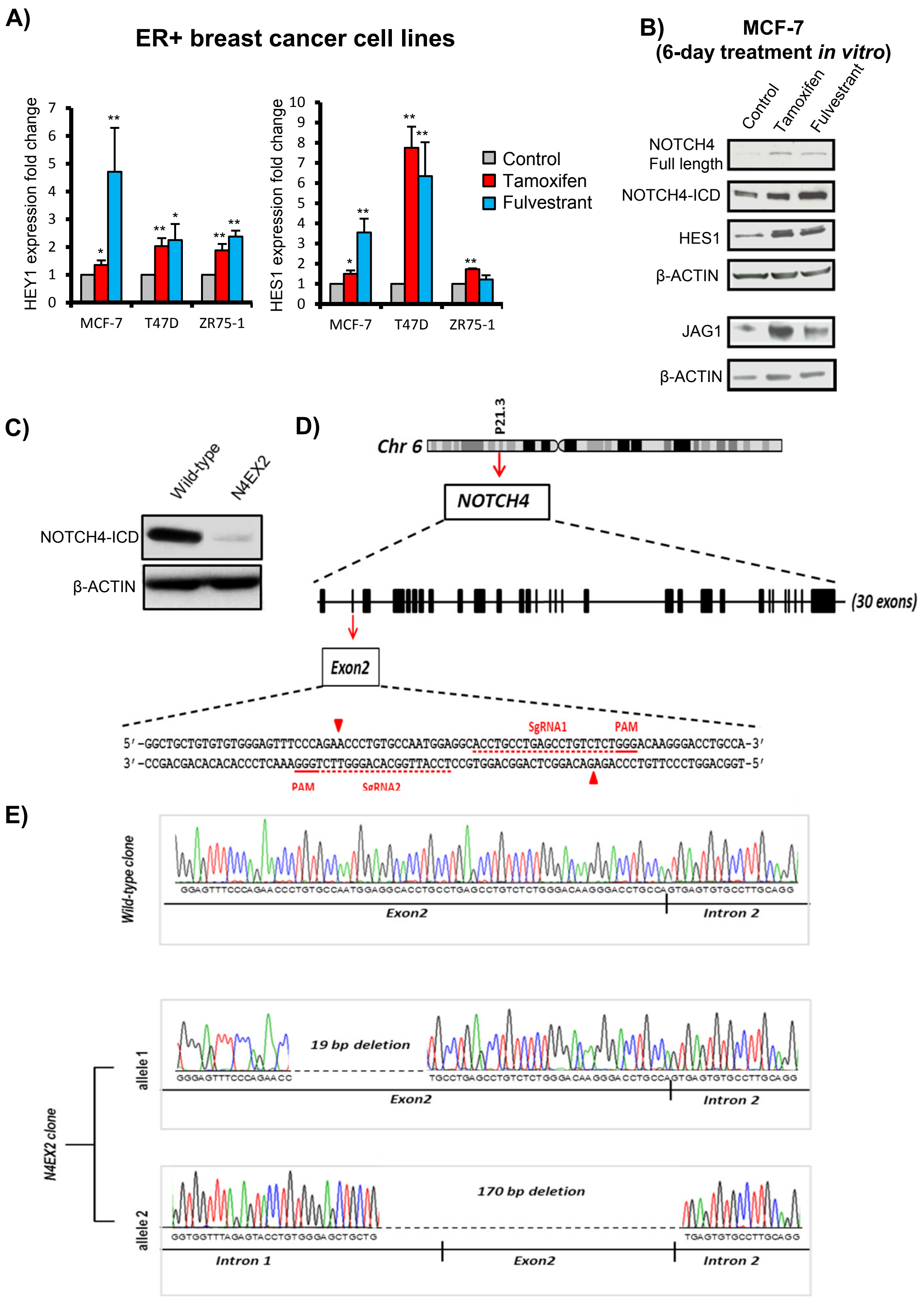

Supplementary figure 3

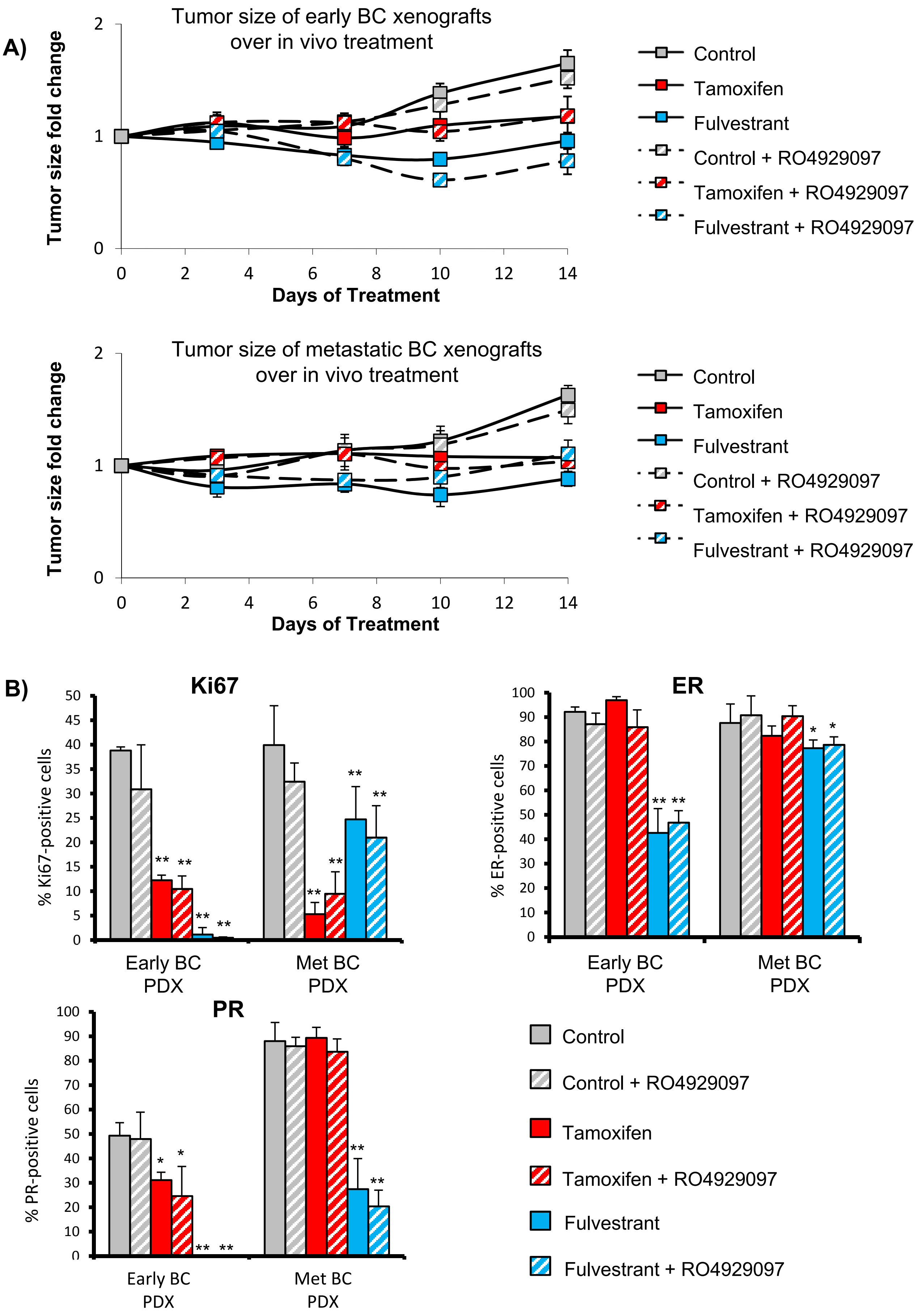

Supplementary figure 4

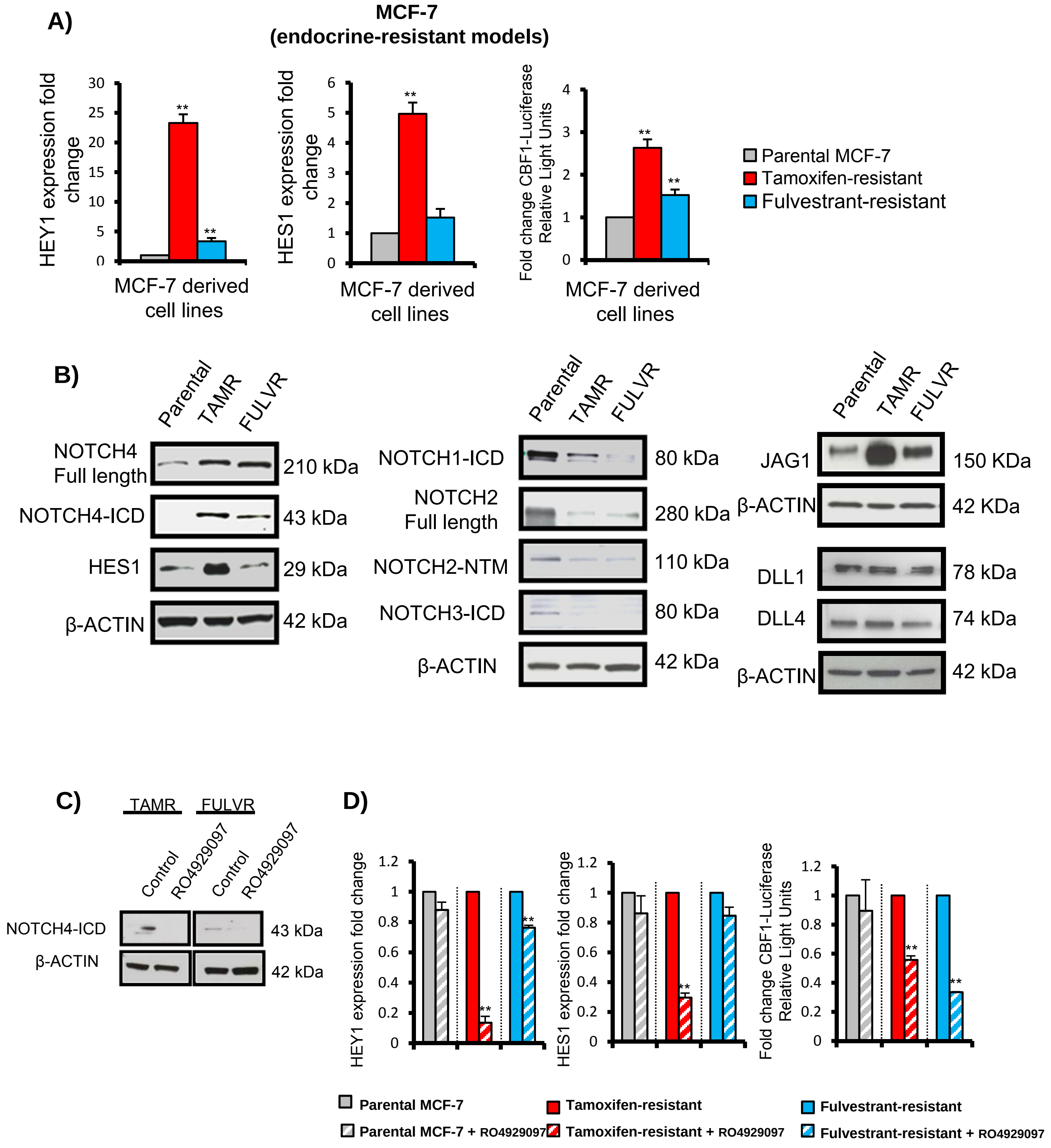

Supplemental Figure Legends

Figure S1, Related to Figure 1:

A) Mammosphere formation efficiency (MFE) of freshly isolated ER+ early and metastatic patient-derived samples cultured in the presence of ethanol (Control) or 10^{-6} M 4-hydroxy-tamoxifen. Mammosphere data for each individual patient sample is represented.

B) Primary mammospheres were dissociated and replated in secondary mammosphere suspension culture for a further 7-9 days to measure self-renewal of mammosphere-initiating cells treated in the first generation.

C) ER positive cells as a percentage of total cell population in parental and aldefluor positive (ALDH+) cells were determined in MCF7 and T47D cell lines by immunocytochemistry. The right hand panel shows representative micrographs of ER staining in MCF-7 cells. Data are represented as mean ± SEM. ** $p < 0.01$

D) Early (HBCx34) and metastatic (BB3RC31) xenograft tumor sections stained for ER, PR, HER2 and Ki67 by immunohistochemistry. Scale bars = 100µm.

E) Ovariectomized mice and administration of estrogen in the drinking water (8µg/ml) were used to perform an estrogen dependence test of metastatic BB3RC31 PDX. Graph shows tumor formation and size at 120 days after implantation. Tumor formation was determined by counting tumors greater than 100 mm3 (tumors bigger than 100 mm3 are represented by solid forms and tumors smaller than 100 mm3 are represented by hollow forms). Mean of each group is represented by horizontal bar. OveX- Ovariectomized; E2 – Estrogen

Figure S2, Related to Figure 2:

A) Expression of Notch target genes *HEY1* and *HES1* was assessed by quantitative real-time PCR analysis and compared to control to determine fold change. MCF-7, T47D and ZR-75-1 cells were treated with 10^{-6} M tamoxifen (red bar) and 10^{-7} M fulvestrant (blue bar) for six days. Data are represented as mean ± SEM. * $p < 0.05$, ** $p < 0.01$

B) NOTCH4, HES1 and JAG1 protein expression levels determined by Western Blot in MCF-7 cells treated with 10^{-6} M tamoxifen (red bar) and 10^{-7} M fulvestrant for 6 days. β-actin was used as a reference for the loading control.

C) NOTCH4 - intracellular domain (ICD) protein levels were determined in wild-type and N4EX2 CRISPR clone by Western Blot. NB: A very small amount of NOTCH4-ICD was detected even though N4EX2 clone contains a frameshift deletion in both alleles, which we hypothesize to be due to a small amount of in-frame RNA produced by whole exon 3 skipping, i.e., by splicing of exon 1 donor site to the acceptor sites of exons that are in-frame.

D) Schematic representation of *NOTCH4* location in chromosome 6 and sgRNA double-nickase CRISPR design to target exon 2 of *NOTCH4*. sgRNAs used are underlined in red dashed line. Predicted cleavage sites for single-strand break start 3 nucleotides upstream of the PAM sequence (underlined in red) and are indicated by the red arrows.

E) Sanger sequencing of wild-type and *NOTCH4* CRISPR targeted clone (N4EX2 clone) showing a deletion of 19 nucleotides in the exon 2 and a deletion of 170 nucleotides, which comprises the entire exon 2.

Figure S3, Related to Figure 3:

A) Early (HBCx34) and metastatic (BB3RC31) PDXs tumor size variation over 14 days in vivo treatments with tamoxifen (10mg/kg/day, oral gavage) or fulvestrant (200mg/kg/week, subcutaneous injection) in the presence or absence of the gamma-secretase inhibitor RO4929097 (3mg/kg/day, oral gavage). Tumor size was determined every 3-4 days and fold change was calculated by dividing the tumor size by the size of the respective tumor at day 0.

B) Quantification of Ki67, ER and PR expression determined by immunohistochemistry. Early (HBCx34) and metastatic (BB3RC31) PDX tumors treated in vivo for 14 days with tamoxifen (10mg/kg/day, oral gavage) or fulvestrant (200mg/kg/week, subcutaneous injection) in the presence or absence of the gamma-secretase inhibitor RO4929097 (3mg/kg/day, oral gavage). Data are represented as mean ± SEM. * $p < 0.05$; ** $p < 0.01$

Figure S4, Related to Figures 2, 3 and 4:

A) Expression of *HEY1* and *HES1* in endocrine-resistant cells compared to the parental MCF-7 cells (two left hand panels). Notch transcriptional activity in endocrine-resistant cells compared to control was determined by relative firefly luciferase activity of 10x CBF1 reporter (right hand panel).

B) Notch receptors and Notch ligands protein expression levels determined by Western Blot in MCF-7 endocrine-resistant cells. β-actin was used as a reference for the loading control.

C) NOTCH4 intracellular domain expression levels determined by Western Blot in MCF-7 endocrine-resistant cells treated with GSI RO4929097 or control DMSO for 72h.

D) Expression of *HEY1* and *HES1* and Notch transcriptional activity (10xCBF1-luciferase reporter) are inhibited by GSI RO4929097 (72h) in endocrine-resistant cells.

Data are represented as mean ± SEM. * $p < 0.05$, ** $p < 0.01$

TABLE S1: Characteristics of 'early' patient-derived tumors used in the study. PBC – primary breast cancer. (Related to Experimental Procedures)

	STAGE	TYPE	GRADE	ER	PgR	HER-2	Neoadjuvant Treatment
PBC 1 (BB2RC5)	Early	IDC	3	+	+	-	-
PBC 2 (BB2RC7)	Early	IDC	3	+	-	+	-
PBC 3 (BB2RC8)	Early	IDC	ND	+	-	-	-
PBC 4 (BB2RC9)	Early	IDC	3	+	+	+	-
PBC 5 (BB2RC10)	Early	IDC	2	+	+	-	Tamoxifen Letrozole
PBC 6 (BB6RC174)	Early	IDC	2	+	+	-	Tamoxifen
PBC 7 (BB6RC180)	Early	IDC	2	+	+	-	-
PBC 8 (BB6RC183)	Early	IDC/ILC	3	+	-	-	-
PBC 9 (BB6RC184)	Early	IDC	3	+	+	-	-

IDC: invasive ductal carcinoma; ILC: invasive lobular carcinoma; ND: not determined

TABLE S2: Characteristics of 'late' metastatic endocrine therapy-treated patient-derived tumors used in the study. Met BC – metastatic breast cancer. (Related to Experimental Procedures)

	TYPE	GRADE	ER	PgR	HER-2	Chemotherapy	Hormonal therapy	Targeted therapy
Met BC 1<br>(BB3RC28)	IDC	1	+	+	-	TOPIC 2 trial<br>Docetaxel Capecitabine	Tamoxifen<br>Exemestane<br>Fulvestrant	Sutent
Met BC 2<br>(BB3RC29)	ILC	2	+	+	-	FEC<br>Paclitaxel<br>Epirubicin	Anastrazole<br>Exemestane<br>Fulvestrant<br>Letrozole	
Met BC 3<br>(BB3RC31)	IDC	2	+	+	-	CMF	Tamoxifen<br>Letrozole<br>Exemestane<br>Low dose Estradiol	Gefitinib
Met BC 4<br>(BB3RC32)	IDC	2	+	+	-	FEC<br>Docetaxel<br>Paclitaxel	Anastrozole	
Met BC 5<br>(BB3RC33)	ILC	2	+	+	-	Epirubicin<br>Capecitabine	Tamoxifen<br>Exemestane<br>Letrozole	
Met BC 6<br>(BB3RC35)	IDC	2	+	+	-	Capecitabine<br>Docetaxel	Letrozole<br>Tamoxifen<br>Goserelin<br>Exemestane	
Met BC 7<br>(BB3RC49)	IDC	2	+	+	-	FEC<br>Paclitaxel	Letrozole<br>Tamoxifen<br>Exemestane	
Met BC 8<br>(BB3RC50)	IDC	2	+	+	-	FEC<br>Paclitaxel<br>Capecitabine<br>Vinorelbine	Tamoxifen<br>Anastrazole<br>Exemestane<br>Fulvestrant	
Met BC 9<br>(BB3RC59)<br>Met BC 9A<br>(BB3RC61)<br>Met BC 9B<br>(BB3RC66)	ILC	2	+	+	-	EOX<br>Capecitabine<br>Paclitaxel	Letrozole<br>Tamoxifen<br>Exemestane<br>Fulvestrant	
Met BC 10<br>(BB3RC68)	IDC	2	+	ND	-	FEC<br>Capecitabine<br>Paclitaxel	Tamoxifen<br>Fulvestrant	
Met BC 11<br>(BB3RC69)<br>Met BC 11A<br>(BB3RC70)	ILC	2	+	+	-	ECF	Tamoxifen<br>Letrozole<br>Anastrozole<br>Fulvestrant	
Met BC 12<br>(BB3RC71)	IDC	3	+	+	+	FEC<br>Capecitabine<br>Vinorelbine<br>Docetaxel<br>Epirubicin	Tamoxifen<br>Anastrozole<br>Fulvestrant<br>Exemestane	Herceptin<br>Lapatinib
Met BC 13<br>(BB3RC77)	ILC	?	+	+	-	Paclitaxel<br>Capecitabine	Letrozole<br>Exemestane<br>Fulvestrant	
Met BC 14<br>(BB3RC81A)	IDC	2	+	+	-	FEC	Tamoxifen<br>Anastrozole	
Met BC 15<br>(BB3RC82)	IDC	2	+	+	-	TAC<br>5-FU	Tamoxifen<br>Anastrozole<br>Letrozole<br>Goserelin	
Met BC 16<br>(BB3RC83)	IDC	3	+	+	-	FEC<br>Docetaxel<br>Vinorelbine<br>Eribulin	Tamoxifen<br>Anastrozole<br>Fulvestrant	
Met BC 17<br>(BB3RC87)	IDC	3	+	+	-	FEC<br>Paclitaxel<br>Capecitabine<br>Epirubicin<br>Eribulin<br>CMF	Tamoxifen<br>Letrozole<br>Fulvestrant	

IDC: invasive ductal carcinoma; ILC: invasive lobular carcinoma; 5-FU: 5-Fluorouracil; FEC: 5-FU, Epirubicin and Cyclophosphamide; FEC-T: 5-FU, Epirubicin , Cyclophosphamide and Docetaxel; CMF: Cyclophosphamide, Methotrexate and 5-FU; TAC: Docetaxel, Doxorubicin and Cyclophosphamide; ECF: Epirubicin,Cisplatin,5-FU; EOX: Epirubicin, Oxaliplatin, Capecitabine; ND: not determined

NB: ER, PgR and HER-2 were assessed in the primary breast cancer tissue sample.

TABLE S3: Follow-up data from tamoxifen vs. control clinical trial. (Related to Figure 1)

Case Processing Summary

aldh12grx	randomiseringsgrupp	Total N	N of Events	CensoredN	Percent
,00	control	90	51	39	43,3%
	tamoxifen	88	27	61	69,3%
	Overall	178	78	100	**56,2%**
1,00	control	83	37	46	55,4%
	tamoxifen	61	26	35	57,4%
	Overall	144	63	81	**56,2%**
Overall	Overall	322	141	181	56,2%

Overall Comparisons

aldh12grx		Chi-Square	df	Sig.
,00	Log Rank (Mantel-Cox)	10,721	1	,001
1,00	Log Rank (Mantel-Cox)	,261	1	,609

Test of equality of survival distributions for the different levels of randomiseringsgrupp.

Supplemental Experimental Procedures

Patient-derived samples

Primary human breast cancer (BC) tissue was collected from patients at South Manchester, Salford Royal and The Pennine Acute Hospitals NHS Foundation Trusts and metastatic human BC tissue was collected at The Christie NHS Foundation Trust. All patients underwent fully informed consent in accordance with local research ethics committee guidelines (study numbers: 05/Q1402/25 and 05/Q1403/159).

Patient-derived xenografts and *in vivo* experiments

For xenografting the BB3RC31 sample a single cell suspension of 1 million tumor cells in mammosphere media (DMEM/F12 media with L-Glutamine (Gibco) containing B27 supplement (Gibco; 12587) and 20 ng/ml EGF (Sigma)) mixed 1:1 with Matrigel (BD biosciences, 356234) was inoculated subcutaneously into dorsal flanks of NSG mice in a volume of 0.2 ml. BB3RC31 is a pleural effusion from a patient who also presented with liver, lung and abdominal metastasis. For further clinico-pathological details of BB3RC31 please see Table S2. The HBCx34 estrogen-sensitive PDX model and the HBCx22 and HBCx34 tamoxifen-resistant PDX models were kindly provided by Dr Elisabetta Marangoni from Institute Curie, Paris (Cottu et al., 2012; Cottu el al, 2014).

To determine tumor initiation capacity of MCF-7 cells or metastatic (BB3RC31) PDX cells treated *in vitro* or *in vivo*, respectively, NSG mice were injected subcutaneously with cells in mammosphere media mixed 1:1 with Matrigel. 90-day slow release estrogen pellets were implanted sub-cutaneously into mice two days before cell injection (0.72 mg, Innovative Research of America). Serial limiting dilution implantation of MCF-7 cells (10,000; 1,000; 100; 10 cells) and the Extreme Limiting Dilution Analysis (ELDA) software (The Walter and Eliza

Hall Institute of Medical Research) were used to perform calculations (95% CI) of tumor initiation. Positive tumor growth was assessed at day 60 or 90 after cell injection by determining the mice bearing a tumor greater than 100 mm^3 and is represented as mice positive for growth/mice tested (n=4 per condition in all experiments). p values were calculated with Chi-squared test.

Cell lines and culture

MCF7, T47D and ZR-75-1 cell lines were purchased from American Tissue Culture Collection (ATCC) and cultured in DMEM/F-12, GlutaMAX (Gibco) with 10% FBS (Gibco). Cells were treated for 3 days or 6 days with ethanol (control), 10^{-6}M 4-OH-tamoxifen or 10^{-7}M fulvestrant and/or with 10µM of gamma-secretase inhibitor RO4929097 (Cellagen Technology) or DMSO (control).

MCF7 Tamoxifen- and Fulvestrant-resistant cell lines were generated by Dr Julia Gee (University of Cardiff, Wales) and were cultured in phenol red-free DMEM/F12 media with L-Glutamine (Gibco) supplemented with 5% charcoal stripped serum and in the presence of 10^{-7}M 4-OH-tamoxifen or 10^{-7}M fulvestrant, respectively (Knowlden et al., 2003; McClelland et al., 2001). MCF7 resistant cell lines were authenticated by comparative karyotyping to ATCC-derived MCF7.

Mammosphere colony assay

Single cell suspensions of freshly isolated ER+ primary and metastatic patient-derived samples were cultured for 7-9 days (primary cells) in mammosphere colony assays in the presence of ethanol, 10^{-6} M 4-hydroxy-tamoxifen or 10^{-7} M fulvestrant. Primary mammospheres were

dissociated and replated in secondary mammosphere suspension culture for a further 7-9 days to measure self-renewal. Patient-derived xenograft cells were cultured for 7-10 days. For cell lines, cells were pre-treated in adherence and then cultured for 5 days in mammosphere culture (200 cells/cm^2 for cell lines).

Aldefluor assay (Stemcell Technologies)

Dissociated single cells were suspended in Aldefluor assay buffer containing an ALDH substrate, bodipyaminoacetaldehyde (BAAA) at 1.5 mM, and incubated for 45 min at 37ºC. To distinguish between ALDH-positive and -negative cells, a fraction of cells was incubated under identical conditions in the presence of a 2-fold molar excess of the ALDH inhibitor, diethylaminobenzaldehyde (DEAB). Mouse cells were excluded from the FACS analysis with anti-mouse MHC Class I (H-2Kd) antibody conjugated with Pacific Blue (BioLegend, 116616). 7-aminoactinomycin D (7AAD, BD) was added for dead cell exclusion. Data were acquired on a LSR II (BD) flow cytometer and analysed using the BD FACSDiva™ software.

Overexpressing MCF-7 cell lines

Overexpression of Notch IntraCellular Domain 4 (NICD4) or Jagged 1 ligand (JAG1) in MCF-7 cells was carried out by lenti- and retro-viral transduction, respectively.

PCR amplified human NICD4 was inserted in pCDH-EF1-MCS-T2A-puro lentiviral vector (System Biosciences), and puromycin resistance gene was replaced by GFP. Lentiviruses were produced by co-transfection of pPsPax2, pMD2.G vectors and the relevant pCDH lentiviral vector (containing NICD4 or empty vector) in HEK293T cells using polyethylenimine

(Millipore). In order to stimulate lentiviral production 10 mM sodium butyrate was added 8-10 h after transfection. Then, virus-containing supernatant was collected after 48h.

Full length human JAG1 cDNA cloned into the retroviral vector LZRSpBMN-linker-IRES GFP was kindly provided by ML Toribio (CBMSO, Madrid). To obtain retroviral particles, the retroviral vectors (hJAG1-expressing vector or the empty vector) were co-transfected with pMD2.G (envelope system) into PhoenixGP packaging cells following the calcium phosphate method (CalPhos Mammalian Transfection kit, Clontech Laboratories). Retroviruses-containing supernatants were harvested 24, 48 and 72h post-transfection.

Once virus-containing supernatants were collected, they were centrifuged at 400 g for 5min and filtered through 0.45 μm filter to remove cell debris. The viral supernatants were concentrated by ultracentrifugation using an AH-629 rotor (Sorvall Ultra Pro80, Sorvall) at 20000 rpm for 2h at 4°C. The viral pellets were resuspended in PBS and aliquots were stored at -80°C. MCF-7 cells were transduced with either lenti- or retro-viral particles in the presence of 8 μg/ml polybrene (Sigma). Stable transduced MCF-7 cells were selected by FACS based on GFP expression.

All cell lines were cultured at 37°C in 5% CO_2 and experiments were carried out at ~70% confluence.

***NOTCH4* targeting using CRISPR-Cas9n technology**

To generate MCF-7 clones with genomic alterations in *NOTCH4*, a double-nickase approach was used by expressing a D10A mutant version of Cas9 (Cas9 nickase) together with a pair of sgRNAs complementary to opposite strands of the targeted genomic locus. sgRNA oligo sequences (sgRNA-A, 5’-ACCTGCCTGAGCCTGTCTCT-3’; sgRNA-B, 3’-TCTTGGGACACGGTTACCT-5’) targeting exon 2 of *NOTCH4* gene were designed using the

CRISPR design tool (http://crispr.mit.edu/) - **Figure S2D**. sgRNA-coding sequences were cloned into pSpCas9n (BB)-2A-GFP (Addgene, PX461) vector. MCF-7 cells were co-transfected with PX461-sgRNA-A and PX461-sgRNA-B plasmids using X-tremeGENE Transfection reagent (Roche, 06366244001). Two days post-transfection, GFP-positive cells were directly FACS sorted as single cells into 96-well plates. After 14 days in culture, clones were screened for CRISPR-mediated indels. Genomic DNA was extracted using the Wizard Genomic DNA Purification Kit (Promega, A1120) and genotyping PCR was performed using a set of primers flanking the CRISPR target site (forward 5'-GGGTACCATGTGGAGAGTGG-3'; reverse 5'-CACAAGAAGCTGGGTGTCAA-3'). PCR products purified with QIAquick PCR Purification Kit (Qiagen, 28104) were cloned into pGEM-T Easy vector and then Sanger sequenced using T7 sequencing primer. Absence of wild-type alleles was confirmed by using a specific restriction enzyme for target sequence (BanI, Biolabs R0118S). Proteins were extracted and separated on 10% SDS-PAGE gel. Membranes were incubated with anti-NOTCH4 (Santa Cruz, sc-5594) and anti-β-actin (Sigma, A1978) antibodies.

Cancer tissue and cells analysis

Tissue microarrays (TMAs) were constructed from all formalin fixed and paraffin embedded tissue. 3 x 1mm cores were taken per tissue block, and TMAs were cut into 3μm thick sections and analysed by immunohistochemistry. Antibodies utilised were anti-ERα (Thermo, SP1), anti-PgR (Dako, M3569), anti-Ki67 (Dako, M7240), anti-HER2 (Vector Laboratories, VP-C380), anti-HES1 (Abcam, ab108937) and anti-ALDH1 (BD Biosciences, 611195). Antigen retrieval was performed either using Target Retrieval Solution pH9 (Dako S2367, for ER, PgR, Ki67 and ALDH1), Target Retrieval Solution pH6 (Dako S1699, for HES1) or in 10mM Citrate buffer (for

HER2) in a 93^{0}C degree water bath for 25 mins. All antibodies were detected using Dako EnVision Detection System Peroxidase/DAB, Rabbit/Mouse (Dako, K5007) and sections were counterstained with haematoxylin. Staining was quantified using Definiens Tissue Studio software. The percentage of positive epithelial cells was scored on 3 cores per tissue sample.

For ER immunocytochemistry, approximately 50000 cells were FACS sorted and then cytospun for 5 min at 80G on poly-lysine coated slides. Cells were fixed with 4% paraformaldehyde (5 min), permeabilised with acetone (4 min) and methanol (2 min), and then blocked for 10 min with Peroxidase Blocking Reagent (Dako, S2001) and further 10 min with 3% goat serum before incubation with primary antibody. ER was then detected as described above for immunohistochemistry.

Gene Expression Analysis Using Quantitative Real-Time PCR

Total RNA was extracted using the RNeasy Plus Mini Kit (QIAGEN, 74104) and the concentration and purity determined using an ND-1000 spectrophotometer (NanoDrop Technologies). Reverse transcription of 1μg of RNA was performed with Oligo(dT) using the TaqMan Reverse Transcription Reagents from Applied Biosystems (N8080234). Samples were incubated on a thermal cycler (MJ Research) for 10 minutes at 25°C, 30 minutes at 48°C and 5 minutes at 95°C. Quantitative real-time PCR reactions were set up in triplicate in 384-well plates and performed on the 7900 PCR machine (Applied Biosystems) using TaqMan® Universal PCR Master Mix (Applied Biosystems) and probes from Universal Probe Library (Roche). Conditions used for amplification of cDNA fragments were as follows: 95°C for 5 min, 40 cycles of amplification – 95^{0}C for 15 sec, 60^{0}C for 1 min. The expression levels were calculated using the

ΔΔCt method and normalised to the housekeeping genes *36B4* and *GAPDH*. The sequences of the primers and probes used can be found in the table below.

Sequences of primers and probes.

GENE	Forward Primer 5' – 3'	Reverse Primer 5' – 3'	Universal Probe Number
HEY1	CGAGCTGGACGAGACCAT	GAGCCGAACTCAAGTTTCCA	39
HES1	GAAGCACCTCCGGAACCT	GTCACCTCGTTCATGCACTC	60
36B4	TCTACAACCCTGAAGTGCTTGAT	CAATCTGCAGACAGACACTGG	6
GAPDH	AGCCACATCGCTCAGACAC	GCCCAATACGACCAAATCC	60

Protein expression analysis using western-blot

Proteins were extracted with Protein Lysis Buffer (25mM HEPES, 50mM NaCl, 30mM NaPP, 50mM NaF, 1% Triton-X-100, 10% Glycerol, 5mM EDTA, Protease Inhibitor cocktail, 1μM PMSF). Lysates were placed on a rotator for 1 hour at 4°C, then were centrifuged at 10000g at 4°C for 10min, and supernatants were collected. Protein concentrations were assessed using the BCA Protein Assay Kit (Thermo Scientific, 23225). Proteins were separated on a 10% gel (Biorad, 456-1033) by SDS-PAGE at 200V for 1 hour, and then were transferred on polyvinylidene difluoride (PVDF) membranes (BioRad, 170-4157) at 25 V/1,300 mA for 15 min using a Trans Blot Turbo (BioRad, 170-4155). Membranes were blocked in a solution of PBS containing 0,05% Tween-20 and 5% skimmed milk (Marvel) for 1h at room temperature and primary antibodies were incubated overnight at 4°C. Primary antibodies used were anti-NOTCH1 (Rockland, 100401405), anti-NOTCH2 (Cell Signalling, D67C8), anti-NOTCH3 (Santa Cruz, sc-7424), anti-NOTCH4 (Abcam, Ab91621), anti-HES1 (Millipore, AB5702), anti-JAG1 (Santa Cruz, sc-6011), anti-JAG2 (Cell Signalling, 2210), anti-DLL1 (Abcam, Ab76655), anti-DLL3 (Abcam, Ab63707), anti-DLL4 (Abcam, Ab7280) and anti-β-actin (Sigma, A1978).

Horseradish peroxidase-conjugated secondary antibodies (Dako, P0447, P0448, P0449) were incubated for 1h at room temperature. Proteins were visualized with Luminata Classico or Luminata Forte (Millipore, WBLUC0100, WBLUF0100) by exposing the membranes to X-ray films (HyperfilmTM MP, Amersham).

Notch Transcriptional assay

To measure the activation of Notch dependent transcription, cells were transfected with CBF1 firefly luciferase reporter (containing 10 copies of a CBF1 consensus sequence) and CMV-Renilla luciferase reporter. Plasmids were incubated with X-tremegene (Roche, 06366244001) in a ratio of 3:1 (µl of X-tremegene:µg of DNA) in OptiMEM (Life Technologies, 11058-021) for 15 minutes, before addition to the culture media. After 48 hours cells were lysed with 1x Passive lysis buffer (5x, Promega, E 1941), put on the rocker for 15 minutes and luminescence was assayed with the Dual-Glo Luciferase assay system (Promega, E2920) following manufacturer's instructions. Luciferase activity was measured using a luminometer (Promega, Glomax Multi+ Detection System with Instinct Software). Luminescence of the firefly luciferase was normalised to that of the renilla luciferase.

Growth assay (SRB assay)

6000 cells were seeded per well in a 96 well-plate at least in triplicate for each condition used. Plates were incubated in a humidified incubator at 37°C with 5% CO_2 and an SRB assay was performed at different time points to assess cellularity. Briefly, cells were fixed with 25µl/well of 50% (w/v) trichloroacetic (TCA) and incubated at 4°C for a minimum of 1hour. Fixed cells were washed 5 times with water and left to air dry. Cells were then stained with 100µl/well of 0.4%

(w/v) Sulforhodamine B (SRB) dissolved in 1% acetic acid for 30 minutes at room temperature. Residual SRB was washed away with 3 washes of 1% acetic acid and plates were left to air dry. Finally, SRB was solubilized with 100µl/well of 10mM Tris-base (pH 10.5) for 20 minutes at room temperature and absorbance was measured at 490 nm with an automated plate reader (BioTek ELx800).

Notch gene expression signature

Gene expression data was summarised with Ensembl alternative CDF (Dai et al., 2005, Nucleic Acids Res 33:e175) and normalised with RMA, before integration using ComBat (Johnson et al., 2007, Biostatistics; 8: 118–127) to remove dataset-specific bias.

References

Cottu, P., Bieche, I., Assayag, F., El Botty, R., Chateau-Joubert, S., Thuleau, A., Bagarre, T., Albaud, B., Rapinat, A., Gentien, D., et al. (2014). Acquired resistance to endocrine treatments is associated with tumor-specific molecular changes in patient-derived luminal breast cancer xenografts. Clinical cancer research : an official journal of the American Association for Cancer Research 20, 4314-4325.

Cottu, P., Marangoni, E., Assayag, F., de Cremoux, P., Vincent-Salomon, A., Guyader, C., de Plater, L., Elbaz, C., Karboul, N., Fontaine, J.J., et al. (2012). Modeling of response to endocrine therapy in a panel of human luminal breast cancer xenografts. Breast cancer research and treatment 133, 595-606.

Dai, M., Wang, P., Boyd, A.D., Kostov, G., Athey, B., Jones, E.G., Bunney, W.E., Myers, R.M., Speed, T.P., Akil, H., et al. (2005). Evolving gene/transcript definitions significantly alter the interpretation of GeneChip data. Nucleic acids research 33, e175.

Johnson, W.E., Li, C., and Rabinovic, A. (2007). Adjusting batch effects in microarray expression data using empirical Bayes methods. Biostatistics 8, 118-127.

Knowlden, J.M., Hutcheson, I.R., Jones, H.E., Madden, T., Gee, J.M., Harper, M.E., Barrow, D., Wakeling, A.E., and Nicholson, R.I. (2003). Elevated levels of epidermal growth factor receptor/c-erbB2 heterodimers mediate an autocrine growth regulatory pathway in tamoxifen-resistant MCF-7 cells. Endocrinology 144, 1032-1044.

McClelland, R.A., Barrow, D., Madden, T.A., Dutkowski, C.M., Pamment, J., Knowlden, J.M., Gee, J.M., and Nicholson, R.I. (2001). Enhanced epidermal growth factor receptor signaling in MCF7 breast cancer cells after long-term culture in the presence of the pure antiestrogen ICI 182,780 (Faslodex). Endocrinology 142, 2776-2788.
